# Unleashing the Potential of Tannic Acid in Dentistry: A Scoping Review of Applications

**DOI:** 10.3390/bioengineering12050438

**Published:** 2025-04-22

**Authors:** Xiaoqian Ding, Guanning Zhang, Cynthia Kar Yung Yiu, Xin Li, Zhiyi Shan

**Affiliations:** 1 Paediatric Dentistry and Orthodontics, Faculty of Dentistry, The University of Hong Kong, 34 Hospital Road, Sai Ying Pun, Hong Kong 999077, China; xiaoqiankq@connect.hku.hk (X.D.); guanning_zhang@connect.hku.hk (G.Z.); ckyyiu@hku.hk (C.K.Y.Y.); 2 Outpatient Department at Longfor Guangnian, The Affiliated Stomatological Hospital of Chongqing Medical University, No.426 Songshi North Road, Yubei District, Chongqing 401147, China; 3 Restorative Dental Sciences, Faculty of Dentistry, The University of Hong Kong, 34 Hospital Road, Sai Ying Pun, Hong Kong 999077, China; xli0712@hku.hk

**Keywords:** tannic acid (TA), dentistry, antibacterial, anti-inflammation

## Abstract

(1) Background: Tannic acid (TA), a water-soluble polyphenol extensively found in numerous plant species, possesses antimicrobial, anti-inflammatory, antioxidant, and adhesive properties. This scoping review aims to synthesize existing knowledge on TA applications and unveil its potential uses in dentistry. (2) Methods: A comprehensive search across six electronic databases (PubMed, Cochrane, Embase, Scopus, Web of Science, and Opengrey) was conducted in October 2024. Two reviewers performed the screening and risk of bias analysis independently following the PRISMA-ScR guidelines. The findings are presented in a narrative summary. (3) Results: Five hundred and twelve records were identified from the electronic databases. After removing duplicates and applying eligibility criteria, ninety-six studies were ultimately included in this review. Results indicate that TA has been employed in managing dentin hypersensitivity, dental caries, periodontal and mucosal diseases, as well as dentition defects with prostheses. Furthermore, TA displays potential in enhancing the performance of bonding adhesives, root canal irrigants, and root canal filling materials. However, it is noteworthy that the included studies exhibit varied experimental settings, inconsistent outcome measures, a lack of extensive clinical research, and insufficient observation periods. (4) Conclusions: TA is a promising biomaterial with applications to various dental fields, such as endodontics, periodontology, prosthodontics, and dental public health. Its antimicrobial, anti-inflammatory, antioxidant, and adhesive properties warrant future exploration to unleash these potentials and provide robust scientific evidence that guides clinical practice and advances oral healthcare.

## 1. Introduction

**Figure 1:**
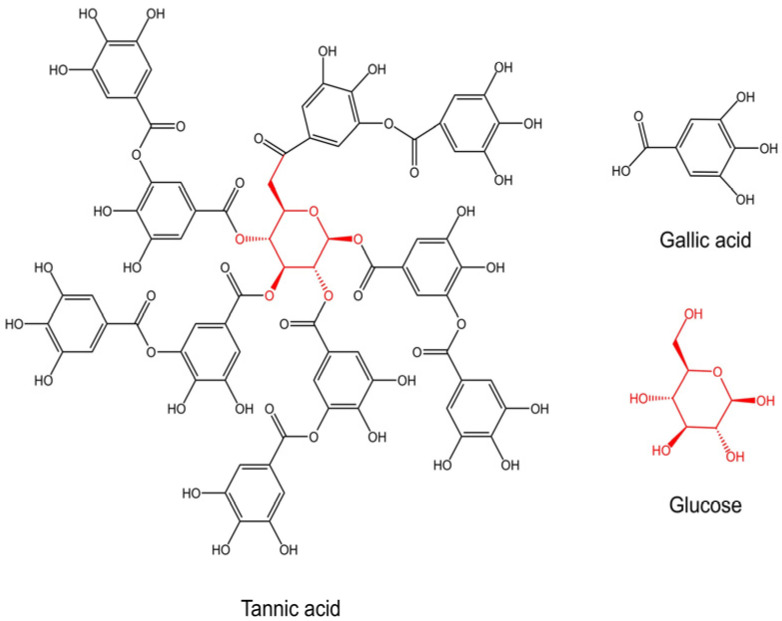
Chemical structure of tannic acid (TA). The red portion represents the core glucose unit.

Tannic acid (TA) is a ubiquitous polyphenolic compound, widely found in a plethora of plant species. Its molecular structure consists of a core glucose unit encircled by ten gallic acid moieties (C_76_H_52_O_46_, Figure 1) [1]. TA has garnered significant attention owing to its diverse chemical properties, which encompass antimicrobial, antioxidant, and anti-inflammatory activities, and has been used in a wide range of medical applications, from preventing infections to promoting tissue regeneration [2,3,4]. These characteristics endow TA with a remarkable degree of versatility, facilitating its comprehensive investigation across multiple domains, including industry, pharmaceuticals, and biomedicine.

The profusion of phenolic hydroxyl groups in TA facilitates robust covalent and non-covalent interactions with natural polymers such as collagen and polysaccharides [5,6]. This compatibility allows for TA functionalization, thereby transforming it into polymers with diverse applications, spanning from wound dressings to metal coatings [7,8]. In the realm of antibacterial traits, TA has demonstrated its ability to compromise bacterial cell membranes, impede bacterial adhesion, and influence nutrient uptake [9,10]. Furthermore, TA’s antioxidant and anti-inflammatory capabilities are evidenced by its ability to reduce oxidative stress and regulate inflammatory mediators, including interleukin-1 (IL-1), IL-6, tumor necrosis factor-α (TNF-α), and prostaglandin E2 [11,12]. These properties have been harnessed in various antiaging, antitumor, cardioprotective, and neuromodulatory therapies, as well as toxicity control measures [11,12,13,14].

In the field of dentistry, TA has been widely explored in the prevention, management, and treatment of oral conditions. These applications include managing dentin hypersensitivity [15,16], arresting dental caries [17,18], and promoting dental implant coating [19,20]. Research also suggests that TA could enhance bond strength and maintain long-term stability when applied to dental surfaces [21,22]. Additionally, TA is capable of withstanding the moist oral environment, making it suitable for non-surgical treatments of periodontal and mucosal diseases [23,24]. Furthermore, TA improves the efficacy of root canal treatments by eliminating residual oxygen produced by sodium hypochlorite [25], as well as by forming complexes with metal ions, such complexes possibly contributing to staining effects [26].

However, despite the promising applications of TA in dentistry, certain limitations or unfavorable outcomes have been reported. A previous study showed that the application of TA did not improve the shear bond strength of a self-adhesive resin to dentin [27]. Another study suggested that the application of TA as a coating for dental implants might inhibit osteoblast activity, as indicated by reduced alkaline phosphatase (ALP) expression and activity, potentially delaying osteoblast maturation [28]. To better understand the breadth and depth of TA applications in dentistry as well as the underlying mechanisms, a comprehensive review of previous studies using an evidence-based approach is highly anticipated. To the best of our knowledge, there is a lack of reviews on the application of TA in the oral field. This scoping review aims to fill this gap by summarizing TA applications across all dental specialties and incorporating narrative insights to discuss the underlying mechanisms and reveal more of its potential and benefits in clinical practice. The research question proposed was “What are the applications and performance of tannic acid (TA) in the field of dentistry?”.

## 2. Materials and Methods

### 2.1. Search Strategy

The scoping review was performed and reported following the Preferred Reporting Items for Systematic Reviews and Meta-Analyses extension for scoping reviews (PRISMA-ScR). An extensive literature search was conducted across six electronic databases (PubMed, Cochrane, Embase, Scopus, Web of Science, and Opengrey), with no publication date restriction. Based on the research question, “tannic acid” and “dentistry” were selected as keywords, together with their synonyms or other related words to avoid omissions following the preformulated search strategy (Supplementary Tables S1 and S2). An updated search was conducted on 31 October 2024.

### 2.2. Study Selection

**Figure 2:**
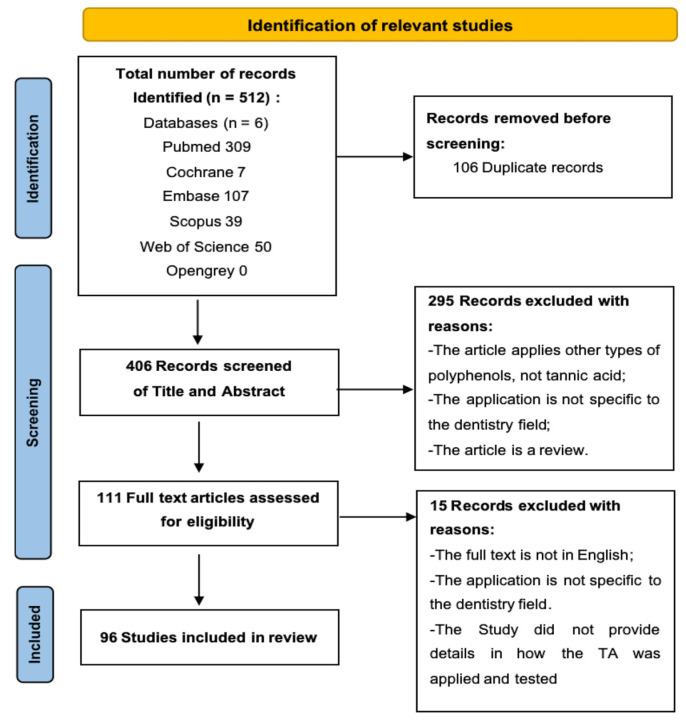
PRISMA flowchart illustrating the study identification, screening, and inclusion process.

All identified articles were imported into EndNote software (version 21, Clarivate Analytics, London, UK). After removing duplicates, two reviewers independently screened the articles based on the following eligibility criteria. Any disagreements between the reviewers were resolved by discussion until consensus was reached. The process is illustrated in the PRISMA flow diagram (Figure 2).

#### 2.2.1. Inclusion Criteria

- Original in vitro studies, in vivo studies, or clinical studies;
- Articles on the application of TA in the field of dentistry;
- Full text in English.

#### 2.2.2. Exclusion Criteria

- Review articles;
- Studies applying other types of polyphenols, not TA.

### 2.3. Data Extraction and Synthesis

Two authors carried out data extraction independently for each of the included studies. The information gathered encompassed the author, year of publication, study design, subjects, interventions (involving TA or its derivatives), assessment methods, and their respective outcomes. All the collected data were then subjected to a thorough analysis and synthesized in a narrative manner to provide a comprehensive understanding of the results.

### 2.4. Quality Assessment

The risk of bias for included studies was determined using an assessment tool adapted from Tran et al. [29]. Nine domains were evaluated: clear objective, suitable study design, clear description of sample size calculation, clear sample dimension, sample randomization, clear intervention methods, TA application with defined concentration(s), appropriate statistical methods, and clear result statements. Each domain received a score of one for clear description or zero for unavailable information. Following evaluation, the studies were classified as being at high (0–4), moderate (5–6), or low risk of bias (7–9).

## 3. Results

### 3.1. Characteristics of Included Studies

There were 512 records identified from the electronic search. Following the removal of duplicates and the screening process based on the eligibility criteria, a total of 96 studies were ultimately included in this scoping review. These studies encompassed seven key aspects in which TA was employed: dentin hypersensitivity management [15,16,30,31,32,33,34,35], bond strength improvement [21,22,27,36,37,38,39,40,41,42,43,44,45,46,47,48,49,50], caries arrest [17,18,51,52,53,54,55,56,57,58,59,60,61,62,63], prosthesis and implant coating [19,20,28,64,65,66,67,68,69,70,71,72,73,74,75,76,77], periodontal and mucosal disease treatment [23,24,78,79,80,81,82,83,84,85,86,87,88,89], endodontic treatment optimization [25,90,91,92,93,94,95,96,97,98,99,100,101], as well as materials for public oral health [26,102,103,104,105,106,107,108,109,110,111].

### 3.2. Quality Outcomes

**Figure 3:**
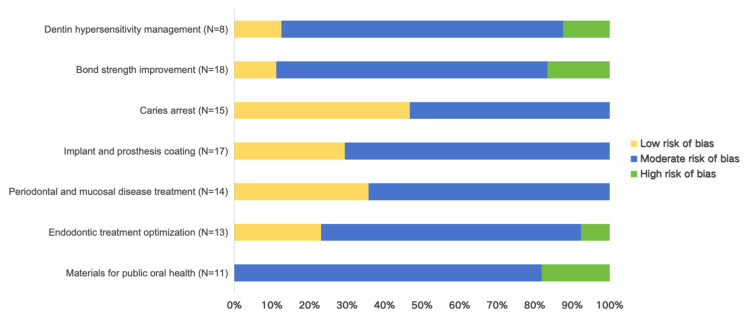
Risk of bias profile of the included studies.

Of the 96 studies included, 23 studies presented a low risk of bias, 66 a moderate risk, and seven a high risk. The results are described in Supplementary Table S3, according to the parameters considered in the analysis. Figure 3 depicts the distribution of high, moderate, and low risk of bias across the different domains. The biases were mainly focused on (1) the lack of precision in the description of the samples, such as the calculation of sample size and the selection of sample dimension; (2) the experimental design limited to in vitro studies, lacking in vivo and clinical study verification; (3) absence of blinding in tests; and (4) unclear descriptions of statistical methods. Additionally, some studies originated from the same research group, potentially leading to some inevitable biases.

### 3.3. Dentin Hypersensitivity Management

Eight articles on dentin hypersensitivity (DH) management were published between 1987 and 2024 [15,16,30,31,32,33,34,35] (Table 1). Three of these studies applied TA alone [15,16,30], while five utilized compounds that combined TA with other agents [31,32,33,34,35].

TA was used to prevent DH by acting on the dentinal tubules. A study by Addy et al. [30] demonstrated that TA, at a pH of 3.3, did not lead to significant dentinal tubule exposure, in contrast to the pronounced effects of sulfuric acid or acidic drinks such as apple juice, wine, and yogurt. Further research conducted by Sabbak et al. [15] reported that the effects of TA on dentinal tubules were both time- and concentration-dependent. Applying TA solutions of 15%, 20%, and 25% on dentin for a period of 5, 10, or 15 min resulted in an increase in the contraction of the dentinal tubule openings proportionally to the increase in the concentration of TA and the application time, suggesting that optimizing the concentration and application time could help manage DH. These tubule-occluding effects have been employed in the development of a commercial TA (≥98%, JZ20140427B, Nanjing Jingzhu Bio-technology Co., Ltd., Nanjing, China), which showed favorable outcomes in sealing the dentinal tubule openings and reducing fluid flow through the tubules [16].

In other studies, TA has been utilized as a crucial component in agents designed to alleviate DH. One such compound, known as “HY”, is a mixture of ZnF2 (50%), SrF2 (25%), TA (20%), and ZnO (5%) [31]. A study found that the addition of HY into glass ionomer cement (GIC) could result in the promotion of dentin mineralization and the contraction of dentinal tubules [31]. The greater the amount of HY incorporated into the GIC, the more pronounced its desensitizing effects. Additionally, other composites containing TA have been developed for DH management. These formulations include fluoride–tannic acid–lanthanum–apatite (FTLA) [32,33], TA and iron ions complexes (TA/Fe^3+^) [34], and SF-TA-DTs (a hydrogel formed by self-assembling silk fibroin (SF) and TA within exposed dentin tubules) [35]; all have been reported to effectively occlude dentinal tubules, reinforce wear resistance, and offer long-term efficacy in DH alleviation.

### 3.4. Bond Strength Improvement

Eighteen in vitro studies explored the effects of TA on bonding preparation and strength enhancement [21,22,27,36,37,38,39,40,41,42,43,44,45,46,47,48,49,50] (Table 2). Published between 1982 and 2023, these studies primarily focused on removing the smear layer and improving the bond strength of dentin and enamel, yet the results remain controversial. Most studies applied TA alone at concentrations ranging from 1% to 50% [21,22,27,36,37,38,39,41,42,43,44,45,46,47,48,49], while two used TA derivatives [40,50].

Studies reported that the effectiveness of smear layer removal induced by TA and the compound was also concentration- and time-dependent [38,39,40,44]. Kapoor et al. [43] found that a 50% TA treatment for 90 s was comparable to a 37% PA treatment for 15 s on the surfaces of enamel, achieving similar bond strength with less enamel dissolution. Moreover, TA could inhibit hydroxyproline release and reverse the high degradability of dentin collagen caused by PA, enhancing its resistance to enzymatic degradation [41]. Natsir et al. [42] confirmed this effect, noting it was time- and concentration-dependent, with stability achieved after 6 h. Furthermore, encapsulating amorphous calcium phosphate within TA and SF nanoshells could enhance the mechanical properties of dentin, stabilize the calcium-to-phosphorus ratio, and promote mineral deposition on dentin surfaces [50].

The effects of TA on the bond strength of dental materials vary with different concentrations and durations. For GIC, one study indicated that a 25% TA treatment of dentin for 30 s was not considered to have a significant impact on the bond strength to GIC [37]. However, the same concentration applied for 60 s enhanced the bond strength of GIC to both enamel and dentin [21,36]. Regarding resin composite, Bedran et al. [22] found that 1 h applications of 10% or 20% TA could improve its bond strength to dentin. Boruziniat et al. [46] reported that bond strengths of resin to dentin treated with 20% or 30% TA for up to 5 min exhibited high bond strength even after a two-month challenge in a collagenase solution. Additionally, research indicated that 5 min treatment with 10% TA [48] or 10 min treatment with 20% TA [49] could mitigate the side effects of bleaching on dentin’s bond strength. Conversely, while some studies indicated that a 20% TA treatment on permanent tooth dentin for 10 min [45] or on primary tooth dentin for 2 min [47] did not enhance resin bond strength. This may stem from TA-induced hydrogen bonds being easily disrupted, limiting interactions with dentin collagen [45]. Furthermore, the lower amount of intertubular dentin in primary teeth may have also contributed to their reduced bond strength [47]. Anil et al. [27] proposed that a 25% TA application for 5 min might reduce resin bond strength by decreasing the availability of Ca^2+^ on the dentin surface, diminishing chemical bonding, while the hydrophilic surface created by TA may hinder interactions with hydrophobic materials like bisphenol and dimbethacrylate, ultimately affecting wettability and adhesion.

### 3.5. Caries Arrest

Upon reviewing fifteen studies published between 1993 and 2024 on caries arrest [17,18,51,52,53,54,55,56,57,58,59,60,61,62,63] (Table 3), nine studies utilized TA alone [17,18,54,55,57,58,59,60,62], and the remaining six combined TA with other compounds [51,52,53,56,61,63]. In terms of study design, six studies were conducted in vitro [18,51,52,54,62,63], five were in situ and ex vivo [17,57,58,59,60], two employed a combination of in vivo and in vitro experiments [56,61], and the remaining two were clinical studies [53,55].

Recent studies have demonstrated the potential efficacy of low TA concentrations (<5%) in caries prevention through various mechanisms. In vitro experiments revealed that 0.4% TA inhibited acid production and biofilm formation in enamel [18], and 0.5% TA treatment on type I collagen increased mechanical properties and enhanced enzyme resistance [54]. Additionally, 1.7% TA was found to reduce the interfacial energy between dentin collagen fibers and minerals [62]. In situ experiments, where volunteers wore bovine enamel or dentin splints rinsed with 1% to 5% TA solution periodically, showed decreased bacterial viability and coverage, thereby inhibiting biofilm formation [17,57,58,59,60]. These findings suggest a promising role of TA in caries prevention. However, it is important to note that a clinical study in Nepal found no additional benefits when combining TA with silver diamine fluoride (SDF) compared to SDF alone [55], indicating the need for further research to fully understand TA’s clinical efficacy in caries prevention.

As a component of composite formulations, Yu et al. [51,52] found that enamel treated with a TA–fluoride mixture exhibited significant deposits of calcium-fluoride-like substances forming multiple binding modes with enamel, including loose attachment, partial integration with enamel crystals, and penetration into enamel pores. The salivary-acquired pellicle bioinspired tannic acid was believed to form nanoscale ultramicrostructures on the enamel surface, thereby increasing its microhardness [56]. The biomimetic mussel-inspired adhesive fluoride system, containing TA, SF, and sodium fluoride, showed excellent biocompatibility and wet adhesion properties while effectively inducing an increase in enamel mineralization density [61]. Phytochemical tannic-acid-mediated biosynthesized gold nanoparticles demonstrated a promising ability to inhibit bacterial growth and biofilm formation [63]. Additionally, research by Yamaga et al. [53] indicates that incorporating HY on the inner denture surfaces in contact with abutment teeth significantly reduced caries prevalence and helped maintain gingival health.

### 3.6. Implant and Prosthesis Coating

Seventeen studies on implant and prosthesis coating were published recently (2018–2023) [19,20,28,64,65,66,67,68,69,70,71,72,73,74,75,76,77] (Table 4), reflecting the rapid advancement in implantology. Researchers have harnessed TA’s biocompatibility, antibacterial, and anti-inflammatory properties, experimenting with various concentrations and immersion times across applications to optimize its performance and efficacy.

TA and its derivatives used in implant and prosthesis coating effectively interact with neighboring tissues, promoting cell adhesion and proliferation [19,28,68,70,72,76,77]. Its antibacterial activity primarily targeted *Escherichia coli* and *Staphylococcus aureus*, efficiently inhibiting bacterial biofilm formation [20,28,64,67,73,76]. However, the inhibitory effect on *Candida albicans* was not significant [71]. TA’s hydrophilicity and adhesive properties improved the mechanical stability of implant coatings [64,67,68,73,76]. Furthermore, TA coatings and hydrogels exert anti-inflammatory and immunomodulatory effects. They suppress the release of inflammatory factors (such as IL-6), reduce reactive oxygen species (ROS) production, modulate macrophage polarization, and activate both complement and coagulation systems [19,28,68,70,71,74,75,76,77]. Conversely, commonly employed chlorhexidine mouthwash and gels offer short-term antibacterial effects through bacterial membrane disruption; however, their prolonged use is significantly constrained by adverse effects such as mucosal irritation and tooth staining [112]. Studies also revealed that TA and its composites promote bone metabolism by enhancing the osteogenic factor expression and inhibiting osteoclast proliferation [19,67,73,74,75,77]. However, Geissler et al. [28] found that TA reduced the expression of osteoblast-related genes, including ALP and osteocalcin, though the specific mechanisms remain unclear.

The disparities in TA concentrations employed for the fabrication of hydrogels and implant coatings were accentuated, and discrepancies emerged among various preparation methodologies. In the case of the gallic-acid-grafted chitosan hydrogel incorporated with TA miniaturized particles, the TA concentration was notably high at 40% [76]. In contrast, the TA concentration in coatings, whether used alone or in composite form, remained relatively low, ranging from 0.01% [20,28] to 2% [68], with no clear correlation to coating time. The immersion time was typically 24 h [28,64,65,66,70,71] or overnight [19] for TA and its composites. UV irradiation could reduce this time to 4 h [68], while vortex mixing required a minimum of 30 s [75]. The layer-by-layer technique for coating preparation involved encapsulating the mixture with a buffer solution, depositing TA and related substances, and performing circulation. The deposition time for TA varied significantly, ranging from 5 min [20] to 4 h [73]. In contrast, preparing an aqueous solution of hydrogen-bonded TA, chlorhexidine acetate, and polyethylene glycol for coating took between 10 and 90 min [72]. Meanwhile, preparing gallic-acid-grafted chitosan–tannic acid miniaturized particle hydrogel required a stirring duration of 12 h [76].

### 3.7. Periodontal and Mucosal Disease Treatment

Fourteen articles published between 1990 and 2003 investigated the application of TA in inhibiting inflammation of periodontal or mucosal tissues [23,24,78,81,82,84,85,87,88,89] and mucosa-associated cancers [79,80,83,86] (Table 5). The majority of these studies employed TA derivatives [23,24,81,82,83,84,85,86,87,88,89], with only three studies utilizing TA alone [78,79,80].

Recognized for its exceptional wet adhesion, anti-inflammatory, and antibacterial capabilities, TA has shown a significant therapeutic potential in oral medicine. Homer et al. [78] demonstrated that low concentrations (0.01%) of TA could inhibit 90% of the proteolytic activity of periodontal pathogens, including *Porphyromonas gingivalis*, *Tannerella forsythia*, and *Treponema denticola*. Additionally, polyvinyl alcohol–tannic acid [81] and polyglutamic acid–tannic acid nanoparticles [88] effectively suppressed *Staphylococcus aureus* and *Escherichia coli*. Spheroids of periodontal ligament stem cells encapsulated within a Fe^3+^-TA coordination network [84] were capable of modulating inflammatory gene expression (such as IL-6 and IL-10) and reducing ROS release. Alginate–silver nanoparticles loaded with TA [82] improved gingival perfusion and enhanced the neurogenic tone of small arteries. In vivo studies using animal models validated the multifaceted capabilities of various TA-based formulations for antibacterial, anti-inflammatory, hemostatic, wet adhesion, sustained drug release, and high tissue-repair properties. These include TA-SF-diclofenac potassium hydrogels [23], gelatin methacrylate–nanoclay–TA hydrogels [85], and SF/TA double-layer microneedle patches [87]. Additionally, chitosan hydrogels incorporating TA and glucose oxidase [24] demonstrated notable glucose sensitivity in diabetic periodontal models. An oral mucosal adhesive formulated from TA and polydopamine-modified zinc oxide [89] was capable of achieving dual-mode real-time regulation through light source switching.

Beyond the above applications, TA is also promising in oral cancer treatment. Darvin et al. [79] discovered that concentrations of TA between 3.4% and 17% induced cell cycle arrest and apoptosis in gingival squamous cell carcinoma, with higher concentrations producing stronger inhibitory effects, possibly by regulating the Jak2/STAT3 pathway. Sheng et al. [80] indicated that low concentrations of TA (0.002% and 0.008%) could mitigate doxorubicin-induced necrosis in normal oral keratinocytes without compromising doxorubicin’s anticancer efficacy. Further research by Ding et al. [83,86] led to the development of isoguanosine-TA hydrogel and manganese-ion-loaded guanosine–tannic acid hydrogel, which not only inhibited the proliferation of dysplastic oral keratinocytes but also exhibited comprehensive antileucoplakia activity.

### 3.8. Endodontic Treatment Optimization

Thirteen articles on endodontic treatment optimization were published between 1989 and 2024 [25,90,91,92,93,94,95,96,97,98,99,100,101] (Table 6). These studies revealed that TA not only improved root canal cleanliness and filling material permeability, but also enhanced the adhesion and sealing ability between materials and dentin.

TA is used as a root canal irrigant and filling material in root canal therapy. Bitter [25] found that rinsing the root canal with hydrogen peroxide or sodium hypochlorite solution, followed by a 25% TA solution, resulted in cleaner and smoother canal walls. This could facilitate the penetration of root canal filling materials. Furthermore, TA irrigation improved the adhesion of filling materials to dentin and their sealing ability [90,91,97]. The novel calcium phosphate bone cement containing TA has been developed as an effective root canal sealing agent, demonstrating suitable setting time and excellent sealing ability [92,95]. When applied to the apical region of mechanically injured molars in rats, this bone cement promoted bone regeneration [93,94]. Furthermore, TA components in HY might help alleviate pulp inflammation [96]. The incorporation of TA into bioactive cements, such as calcium-silicate-based cements, enhance their mechanical and biological properties [98,99]. Additionally, recent research explored the incorporation of TA into a hydroxypropyl chitin hydrogel as a novel pulp capping material [101]. This hydrogel exhibited strong antibacterial properties and effectively enhanced the proliferation of human dental pulp cells while suppressing pro-inflammatory cytokines, highlighting its promise for vital pulp therapy. Apart from root canal treatment, TA shows promise in regenerative endodontics. Louvrier et al. [100] developed a polylactic acid/polycaprolactone-TA using electrospinning/electrospraying techniques. This biomaterial improved the adhesion and proliferation of dental pulp stem cells and simulated the fluid dynamics of residual apical blood vessels.

### 3.9. Materials for Public Oral Health

Eleven articles published between 1983 and 2023 explored TA applications in various public oral health materials [26,102,103,104,105,106,107,108,109,110,111] (Table 7). These studies explored the application of TA in ultrastructural observations [102,103], fixing techniques [104,105], dental staining [26,106,107,108,109], and cleaning agents [110,111].

Kageyama et al. [102] demonstrated that TA–uranyl acetate could reveal the structure of odontoblasts, preodontoblasts, and dentin matrix. Similarly, Takagi et al. [103] employed this method to examine the relationship between basement membrane and collagen fibrils in the predentin. TA has also been utilized for the fixation of enamel and dentin cells and tissues [104,105]. Additionally, a study by Nordbö et al. [26] reported that using TA alone did not cause discoloration, but reactions between chlorhexidine or iron ions and TA might have led to color precipitation, with different concentrations resulting in different colors. The interaction between TA and salivary proteins might be a key step in the staining process [106]. Leveraging this property, researchers used TA as artificial stains for exploring the effectiveness of oral hygiene tools, such as toothbrushes and toothpaste in removing dental plaque [107,108,109]. Interestingly, TA also functions as a component in some cleaning agents for stain removal. Asghar et al. [110] suggested that TA might reduce staining and discoloration caused by SDF. A transparent solution containing TA and the antibacterial compound cetylpyridinium chloride effectively reduced bacterial biofilm and stains on clear aligners [111].

## 4. Discussion

Through the review of TA applications in dentistry, it was established that TA and its compounds serve multiple purposes in dentistry, not only enhancing the mechanical properties of tooth structures, but also managing oral diseases through biological properties with pathogens and the host environment to alleviate inflammation and modulate cell metabolism. In vitro and in situ studies demonstrated that TA positively affects enamel and dentin surfaces by influencing microhardness [54], microstructures [32,33], and acid resistance [52]. These benefits likely stem from TA’s polyphenolic structure, which forms complexes with calcium ions, promotes mineral deposition, and creates stable crosslinked structures with collagen, ultimately enhancing enamel hardness and dentin compressive strength while slowing demineralization [17,18]. The biological reactions were primarily reflected through TA’s antibacterial [67,68] and anti-inflammatory properties [76,77], along with its roles in bone metabolism regulation [73] and cancer impacts [80]. Key indicators include the inflammatory factor expression (IL-6, IL-10, and TNF-α) [84,101], ROS regulation [76,77], and macrophage polarization [74,75]. Studies have also examined TA’s inhibitory effects on oral pathogens, including cariogenic *Streptococcus mutans* [64] and periodontitis-related *Porphyromonas gingivalis* [76]. While Weber et al. found that TA alone did not affect *Candida albicans* [71], its composite, together with zinc oxide, methacrylate gelatin, and methacrylate hyaluronic acid (ZPTA-G/HMA), exhibited antimicrobial effects against *Candida albicans* [89].

Among various natural extracts, TA, as a high-purity form derived from gall nuts, stands out due to its core advantage stemming from a rich content of gallic-acid-derived pyruvate glucosides (GGs), which constitute 20% to 30% of its dry weight [18]. In contrast, conventional gall nut water extracts contain less than 5% GGs [57]. TA exerts a multifaceted antibacterial action through synergistic mechanisms—including membrane disruption, enzyme inhibition, and signaling interference—effectively targeting cariogenic bacteria [113]. Its efficacy in inhibiting biofilm formation of *S. mutans* surpasses that of propolis and gall nut extracts [57]. Furthermore, TA significantly enhances the bond strength between resin and dentin while substantially reducing microleakage, outperforming other natural crosslinking agents such as proanthocyanidins [16]. These attributes position TA as a versatile natural component that integrates efficient antibacterial properties, active mineralization, and material modification, whereas other extracts typically offer only single or dual functionalities. In addition to plant extracts, various emerging technologies are being explored for the prevention and treatment of oral infections and inflammatory diseases, such as Er:YAG laser and photodynamic therapy [114,115]. These methods selectively target bacteria through photothermal effects, modulate inflammatory factors, and promote soft-tissue healing [116,117]. However, they face challenges such as high equipment dependence, elevated costs, risks of thermal damage, and poor sustained antibacterial effects, all of which limit their clinical application [118,119]. In the future, through technological innovation and exploration of alternative mechanisms, it is expected that TA can be combined with new technologies to optimize strategies for the prevention and treatment of oral diseases.

Interestingly, we found that many of the included studies were conducted in the last century. Early research provided a foundational understanding of TA, including its chemical properties, biological activity, and potential applications. These classic studies have laid an important theoretical foundation and provided experimental data for modern research. Concentrations of TA used in dentistry vary significantly, ranging from 0.000001% [78] to 50% [43]. Higher TA concentrations are predominately utilized in the treatment of tooth surfaces for their acidic properties. Research indicates that higher TA concentrations, combined with longer application times, result in more pronounced erosive effects on tooth surfaces [15,22,24]. Conversely, lower TA concentrations are more favorable for chemical reactions, pH regulation, and antioxidant properties, all of which are necessary for ion interactions [18,70,71]. In recent years, an increasing number of studies have explored TA as a composite component, investigating its deeper applications across various fields, further reflecting the ongoing interest and exploration in TA research. When incorporated into composite materials, TA concentrations also exhibited significant variability, ranging from 0.02% [89] to 50% [81]. Given that interactions among composite components could produce substances potentially detrimental to cellular vitality, thorough cytotoxicity evaluation is essential prior to intraoral application of TA composite materials. It is encouraging to notice that current not only emphasize the success rates of material synthesis and characterization, but also ensure, to some extent, the biocompatibility of the composites. These characteristics highlight the significance of adopting proper TA concentrations while ensuring its biological safety for diverse applications in dental research and practice. Yet few studies have systematically investigated the optimal concentration ranges for specific clinical applications or established standardized protocols for TA usage across different dental procedures.

As a component of composite materials, TA excels in potentially creating synergistic effects that enhance the overall performance of metal ions [34,73], fluoride compounds [51,52,61], antibacterial agents [67,69,72,73], and proteins [20,35,87]. This may be attributed to the phenolic hydroxyl groups of TA, which enable its easy connection with other components, thereby improving the stability and biocompatibility of metal ions [120,121], stabilizing fluorides to increase their local concentrations [122,123], as well as modifying the active sites of lysozymes to enhance its ability to disrupt bacterial cell walls and inhibit bacterial growth [10]. However, despite the above benefits of TA in composites, specific biological reactions still require further exploration. Current research has mainly focused on the chemical properties of TA and its interactions with other molecules, while the actual impacts of these complexes within biological systems remain relatively underexplored. This gap might limit the understanding of TA’s potential applications in the oral context. Furthermore, TA’s effects on bone metabolism are unclear, with current studies lacking comprehensive investigations and exhibiting some inconsistencies across different research efforts [28,73,74,75]. Unveiling TA’s mechanisms and uncovering its potential biological effects of action, with consideration of its interactions with other compounds, would aid TA applications in better bone healing and regeneration for periodontitis and implant patients.

Some limitations in TA dentistry research present opportunities for further exploration. First, varied experimental settings highlight heterogeneity in research subjects and application conditions. Differences in TA concentration, application time, delivery method, and sample selection (e.g., human vs. bovine teeth) and treatment are evident. While most studies detail sample preparation, some lack this information [38,39]. Similarly, prosthetic applications use diverse materials like titanium, silicon dioxide, and polymethyl methacrylate, and outcomes are measured using inconsistent metrics, hindering cross-study comparability [20,70,71,72]. Clear guidelines for research subjects, application conditions, and consistent efficacy metrics are needed. Second, the lack of in vivo and clinical validation is a significant shortcoming. Most studies are conducted in controlled labortory settings, with fewer than half including in vivo or clinical models [35,50]. While in vitro experiments provide preliminary efficacy data, they may not reflect real physiological complexities. Future research should prioritize well-structured in vivo and clinical studies, particularly randomized controlled trials (RCTs), to ensure the applicability of the findings to patient care. Lastly, most studies focus on TA’s short-term effects, lacking long-term analysis, particularly in implant coatings and hydrogels [76,77]. While promising in vitro, factors like material degradation, biochemical interactions, and property changes over time could impact long-term efficacy and implant prognosis. More long-term studies are urgently needed to verify TA’s stability and safety in real oral environments.

## 5. Conclusions

In summary, TA, as a plant-derived natural polyphenol, has broad applications in dentistry, including the following:

- Management of dentin hypersensitivity
- Improvement of bond strength
- Caries arrest
- Coating for prostheses and implants
- Treatment of periodontal and mucosal diseases
- Optimization of endodontic treatment
- Materials for public oral health

However, the included studies exhibit varied experimental settings, inconsistent outcome measures, a lack of clinical evidence, and insufficient observation periods. Future research should focus on investigating TA’s long-term effects, exploring its mechanisms and biological responses in oral environments, and conducting high-quality RCTs to assess its efficacy and safety in various dental applications. Through continued research and innovation, TA shows promise for advancing oral health and broader medical applications.

## Figures and Tables

**Table 1:** Summary of applications of TA and its derivatives in dentin hypersensitivity management.

Studies	Treatment	Study Type	Subject	Assessment	Key Outcomes
Addy et al. (1987) [30]	TA (pH 3.3)	In vitro	Human dentin	TA application for 5 min	Nitric, sulfuric, citric, and lactic acids (pH 0.6–2) expanded dentinal tubules. TA (pH 3.3) and formic acid (pH 3.8) had no significant impact on dentin surface. Temperature increase from 25 °C to 37 °C did not affect the opening of dentinal tubules by the acids used.
Yamaga et al. (1993) [31]	HY (20% TA)	In vitro	Bovine dentin	HY application with GIC	Incorporation of HY into GIC promotes CaF2 formation. Increasing HY content (0%, 1.5%, 5%, 10% by weight) resulted in a greater resistance increase and dye penetration inhibition.
Sabbak et al. (1998) [15]	15%, 20%, and 25% TA	In vitro	Human dentin	TA application for 5–15 min	Increased TA concentrations (15–25%) and exposure time (5–15 min) significantly enhanced smear layer removal and dentinal tubule occlusion. Maximum reduction in dentinal tubule openings observed at 20% and 25% TA concentrations after 10 and 15 min of application.
Mukai et al. (1998) [32]	FTLA (5% TA)	In vitro	Bovine dentin	FLTA application for 6 weeks	Increased quantity of small spherical deposits in dentinal tubules. Dentinal tubules occluded to a depth of approximately 3 μm. Fluoride ions diffused into intertubular dentin, with lanthanum and aluminum ions similar to predemineralization measurements.
Tomiyama et al. (2004) [33]	FTLA (5% TA)	In vitro	Bovine dentin	FLTA treatment and toothbrush abrasion test (6000 cycles)	After 6000 wear tests, the tubules remained occluded. Fluoride, lanthanum, and aluminum ions detected on the dentin surface.
Oh et al. (2015) [34]	TA/Fe^3+^ (0.04% TA)	In vitro	Human dentin	TA/Fe^3+^ treatment and toothbrush abrasion test (1000 cycles)	TA/Fe^3+^ composite film occluded dentinal tubules to a micron-level thickness within 4 min. The dentin sealing effect resisted mechanical brushing stimuli. TA/Fe^3+^ composite demonstrated superior efficacy compared to TA alone.
Li et al. (2020) [16]	Commercial TA (≥98%)	In vitro	Bovine dentin	Triple TA application (5 min each)	Average area (2.27 ± 1.7 μm^2^), diameter (1.58 ± 0.62 μm), and occlusion rate (56.02 ± 32.95%) in the TA group were significantly lower than those in the control group (4.88 ± 2.22 μm^2^, 2.43 ± 0.57 μm, and 5.3 ± 43.04%, respectively).
Gao et al. (2024) [35]	SF-TA-DTs (10% TA)	In vitro and in vivo	Human dentin, rat model, rabbit model	SF-TA-DTs application and in vivo evaluation in rat and rabbit models	SF-TA-DTs hydrogel penetrated over 600 μm in depth. Dentin sealing effect resisted mechanical brushing stimuli. SF-TA-DTs exhibited stronger dentinal tubule occlusion than SF or TA alone. SF-TA-DTs showed good proliferation characteristics for hDPSCs and hGF, with no significant inflammatory response or cell damage in rat tongues, cheeks, and palates. In vivo studies in rabbits demonstrated complete dentinal tubule occlusion.

TA, tannic acid; HY, tannin–fluoride; FTLA, fluoride–tannic acid–lanthanum–apatite; Fe^3+^, iron(III) ion; SF-TA-DTs, silk fibroin–tannic acid–dentin tubules; GIC, glass ionomer cement; hDPSCs, human dental pulp stem cells; hGF, human gingival fibroblasts.

**Table 2:** Summary of applications of TA and its derivatives in bond strength improvement.

Studies	Treatment	Study Type	Subject	Assessment	Key Outcomes
Powis et al. (1982) [36]	25% TA	In vitro	Human enamel and dentin	TA application for 60 s, bonded with GIC	TA treatment resulted in smooth enamel and dentin surfaces without etching. TA group bonding strength: enamel (7.02 MPa) vs. control (3.18 MPa, *p* < 0.05). Dentin bonding strength: TA (6.32 MPa) vs. control (3.13 MPa, *p* < 0.05), increased to 7.3 MPa after 72 h.
Prati et al. (1989) [37]	25% TA	In vitro	Human dentin	TA application for 30 s, bonded with GIC	Dentin bonding strength: TA (34.17 ± 4.64 kg/cm^2^) vs. control (38.90 ± 17.67 kg/cm^2^, *p* > 0.05). Dye penetration along dentin walls: TA (0.465), comparable to control (0.460). Dye penetration into the pulp chamber: TA (0.091) vs. control (0.031).
Bitter (1989) [38]	25% TA	In vitro	Human dentin	TA application for 15, 30, and 60 s	TA treatment improved dentin cleanliness, with no pulp response. Extended exposure time (15–60 s) and enhanced cleaning before bonding.
Bitter (1990) [39]	25% TA	In vitro	Human dentin	TA application for 15 s	Dye penetration along dentin walls: TA (62 samples: 48 no, seven slight, two moderate, five severe) vs. control (64 samples: nine no, two slight, 17 moderate, 36 severe).
Okamoto et al. (1990) [40]	HY (20% TA)	In vitro	Human dentin	HY application with GIC intraoral and teeth extraction performed after 2–9 months	HY adhesive showed close contact with unetched dentin; control gaps ranged from 5 μm to 60 μm. Bond strength of HY with dentin and resin showed no significant change over 2 to 9 months. Fluoride and zinc penetrated dentin up to 1500 μm in depth, with increased penetration over time.
Okamoto et al. (1991) [41]	1% TA	In vitro	Bovine dentin	PA application followed by TA treatment for 5, 10, 30, 60, and 120 min	The 7 M PA pretreatment dissolved over 85% of dentin collagen. TA treatment reduced collagen dissolution to 75% (5 min) and 20% (30 min). After 1 min of PA, TA reduced dissolution to 28% (5 min) and 12% (30 min). TA restored collagen resistance to trypsin digestion after 2 h.
Prati et al. (1992) [21]	25% TA	In vitro	Human dentin	TA application for 60 s, bonded with GIC	In 5 min bonding tests, the TA group’s bonding strength (6.21 ± 1.41 MPa) was higher than that of the control group (4.36 MPa, *p* < 0.05). In 24 h tests, the TA group’s bonding strength (8.02 ± 2.6 MPa) exceeded that of the control group (5.94 MPa, *p* < 0.05). TA treatment resulted in less acid etching, with minimal etching of peritubular dentin matrix.
Natsir et al. (1999) [42]	1%, 3%, 5%, and 10% TA	In vitro	Bovine dentin	TA application for 1, 3, 6, 12, or 24 h, followed by PA treatment with trypsin	Increasing TA concentration from 1% to 10% reduced hydroxyproline to about 60%. At 5% TA, hydroxyproline release decreased to 50% over 1 to 24 h. TA pretreatment for 6 h, followed by 40% PA treatment, preserved dentin collagen structure.
Kapoor et al. (2002) [43]	50% TA	In vitro	Human enamel	TA application for 60 s and 90 s, bonded with brackets	TA treatment produced more uniform etching patterns than phosphoric acid. Bonding strength after 50% TA for 90 s (9.09 ± 1.93 MPa) was comparable to 37% phosphoric acid for 15 s (9.10 ± 1.95 MPa, *p* > 0.05). Significantly higher than 50% citric acid treatment for 60 s (5.82 ± 1.39 MPa, *p* < 0.05).
Buchalla et al. (2007) [44]	5% TA	In vitro	Bovine dentin	TA application for 20 s	TA partially cleansed the smear layer from the dentin surface. Fluoride uptake in EDTA or phosphoric-acid-treated dentin was lower than in TA-pretreated layers.
Bedran-Russo et al. (2009) [22]	1%, 10%, and 20% TA	In vitro	Human dentin	TA application for 10 min, 30 min, 1 h, 2 h, 24 h, and 48 h, bonded with resin	Demineralized dentin pretreated with TA for 1 h showed significant increases in ultimate tensile strength: control (8.94 ± 4.03 MPa); 1% TA (13.87 ± 4.26 MPa); 10% (14.87 ± 4.87 MPa); 20% (16.93 ± 5.79 MPa, *p* < 0.05). After 24 h incubation with bacterial collagenase, the control’s ultimate tensile strength dropped to zero, while TA-treated specimens remained stable.
Pavan et al. (2010) [45]	20% TA	In vitro	Human dentin	TA application for 10 min, bonded with resin	TA partially cleansed the smear layer from the dentin surface. TA application did not significantly affect microtensile bond strength (RelyX Unicem resin: control: 8.35 ± 1.99; TA: 8.38 ± 2.67; Maxcem Elite Resin: control: 8.45 ± 3.21; TA: 6.89 ± 4.45, *p* > 0.05).
Anil et al. (2015) [27]	25% TA	In vitro	Human dentin	TA application for 5 min, bonded with resin	Shear bond strength of self-adhesive resin in the TA group (6.57 ± 4.15 MPa) was lower than that in the control group (12.18 ± 3.90 MPa, *p* < 0.05). Both TA and control groups exhibited adhesive failure.
Alireza et al. (2017) [46]	20% and 30% TA	In vitro	Human dentin	TA application for 30 s, 1 min, 3 min, and 5 min, bonded with resin	In immediate tests, bonding strength in TA group (9.970 ± 3.04 MPa) was comparable to control (10.703 ± 2.59 MPa, *p* > 0.05). After 2 months in collagenase solution, 20% the TA group’s bonding strength (7.985 ± 1.754 MPa) exceeded that of the control group (5.285 ± 1.020 MPa, *p* < 0.05). Adhesive failure was the most common failure mode across all groups.
Abdollahi et al. (2017) [47]	20% TA	In vitro	Human dentin	TA application for 2 min, bonded with resin	For Adper single bond, bonding strength in the TA group (30.2 ± 11.1 kg/cm^2^) was lower than that in the control group (63.01 ± 23.75 kg/cm^2^, *p* < 0.05). After aging, strength in the TA group increased (33.38 ± 21.41 kg/cm^2^, *p* > 0.05). For clearfil SE bond, the TA group’s bonding strength (70.68 ± 16.23 kg/cm^2^) exceeded that of the control group (60.39 ± 18.91 kg/cm^2^, *p* < 0.05). After aging, TA strength decreased (51.6 ± 17.81 kg/cm^2^, *p* > 0.05). Adhesive failure was predominant in both groups.
Cecchin et al. (2018) [48]	10% TA	In vitro	Human dentin and root canal	NaOCl application followed by TA treatment for 5 min, bonded with resin	For etch-and-rinse adhesive (single bond), the TA group’s bonding strength (53.86 ± 10.38 kg/cm^2^) was slightly higher than that in the control group (52.34 ± 9.48 kg/cm^2^, *p* > 0.05). For self-etch adhesive (Scotchbond Universal), the TA group’s bonding strength (46.43 ± 12.38 kg/cm^2^) was lower than that of the control group (55.00 ± 8.89 kg/cm^2^, *p* < 0.05).
Shafeie et al. (2022) [49]	20% TA	In vitro	Human dentin	Internal bleaching followed by TA application for 10 min, bonded with resin	TA partially reversed the impact of bleaching agents on bonding strength. Duolink—control: 16.19 ± 1.6 MPa; bleached: 12.52 ± 1.54 MPa; and TA: 13.27 ± 1.8 MPa. Panavia SA—control: 11.8 ± 1.60 MPa; bleached: 1.58 ± 0.62 MPa; TA: 6.12 ± 1.23 MPa.
Zheng et al. (2023) [50]	ACP@TS (0.8% TA)	In vitro and in vivo	Human dentin, rat model	ACP@TS treatment and in vivo evaluation in rats	ACP@TS accumulated on dentin surfaces, penetrating the subsurface (about 15 microns) within 5 min. An oversaturated ionic environment promoted mineral precursor conversion to hydroxyapatite, resisting brushing and EDTA erosion. Dentin treated with ACP@TS partially restored mechanical properties weakened by EDTA etching (0.66 ± 0.05 GPa). No cytotoxicity was observed. After 14 days in the oral cavity of rats, ACP@TS-treated dentin’s Vickers hardness returned to healthy levels (0.75 ± 0.10 GPa).

ACP@TS, amorphous calcium phosphate–tannic acid–silk fibroin; PA, phosphoric acid; NaOCl, sodium hypochlorite.

**Table 3:** Summary of applications of TA and its derivatives in caries arrest.

Studies	Treatment	Study Type	Subject	Assessment	Key Outcomes
Yu et al. (1993) [51]	TA-F (0.5% TA)	In vitro	Human enamel	Rinsed after treatment with TA-F	TA-F deposits on the enamel surface, approximately 2.5–5 μm in height, exhibit strong acid resistance. Significant increases in fluoride and calcium observed within the outer 20 μm.
Yu et al. (1995) [52]	TA-F (1% TA)	In vitro	Human enamel	Rinsed after treatment with TA-F	Acid resistance of TA-F reached 98%. Calcium release inhibition rate after 5 min of treatment was about 50%, sharply increasing to over 90% after 4 h. Fluoride concentration significantly increased after 1 h.
Yamaga et al. (1997) [53]	HY (20% TA)	Clinical	Exposed dentin of overdenture abutments	Denture base mixed with HY	Caries rate in the HY group (13.8%) was lower than that in the conventional resin group (75%). No differences were observed in gingival recession.
Koide et al. (1997) [54]	0.5% TA	In vitro	Type I dentinal collagen from bovines	TA application for 5 min, 1 h, and 1 day	Tensile strength in the 1 h TA treatment group (48.5 ± 3.8 MPa) was significantly higher than that in the control group (37.7 ± 4.5 MPa, *p* < 0.05). Elongation at break increased with treatment time in 5 min and 1 h TA groups. Elastic modulus was lower in the 5 min and 1 h groups compared to the control group, while it increased in the 1 day group. Enzyme activity release was delayed.
Yee et al. (2009) [55]	TA (Mechhi Tea)	Clinical	Primary teeth	Applied to tooth surface with SDF	No significant difference were found in the average number of arrested carious lesions between the 38% SDF group and the 38% SDF + tannic acid group over a 24-month study period.
Yang et al. (2017) [56]	SAP-TA (1.7% TA)	In vitro and in vivo	Human enamel, rat caries model	SAP-TA added after enamel demineralization	Strongly adsorbs onto HAP slice surfaces and exhibits acid resistance. Treated crystals showed an ordered nanorod structure, effectively restoring microhardness. Adhesion of newly formed hydroxyapatite on the enamel surface in the SAP-TA/Fe(III) treatment group (64.85 N) was greater than that in the SAP-TA group (62.95 N) and control group (0.1 N). Low cytotoxicity. In vivo experiments showed significant remineralization effects.
Hertel et al. (2017) [17]	1.7% TA	In situ and ex vivo	Enamel from bovines worn by volunteers as maxillary splints	TA application for 10 min	TA rinsing significantly reduced calcium and phosphate loss. Biofilm formation electron density increased. After 8 h, TA rinsing significantly reduced bacterial adhesion on the enamel surface compared to chlorhexidine.
Huang et al. (2017) [18]	0.4% TA	In vitro	Bovine enamel	TA application for 5 min in pH-cycling and polymicrobial biofilm model	TA inhibited acid production and biofilm formation in composite microbial biofilms. Calcium depletion rates for TA were lower than those for the Galla chinensis extract, showing stronger antidemineralization capability.
Xi et al. (2020) [57]	1% TA	In situ and ex vivo	Enamel from bovines worn by volunteers as maxillary splints	Rinsed with TA twice	Both TA and Chinese gallnut extracts significantly reduced biofilm formation on the enamel and dentin after 24 h compared to the control (*p* < 0.05). No significant difference was observed between the two experimental mouthwashes (*p* > 0.05).
Schestakow et al. (2020) [58]	5% TA	In situ and ex vivo	Enamel from bovines worn by volunteers as maxillary splints	Rinsed with TA for four or five times	Rinsing with water or hydrochloric acid resulted in bacterial coverage rates of 47–55%. Rinsing with TA (pH = 2.5), TA (pH = 7), or chlorhexidine significantly reduced coverage rates to 2–11% (*p* < 0.05). Most samples were covered by a pellicle after TA or chlorhexidine application, with some covered by individual bacterial colonies, mainly rod-shaped and cocci bacteria.
Schestakow et al. (2021) [59]	5% TA	In situ and ex vivo	Dentin from bovines worn by volunteers as maxillary splints	Rinsed with TA for four or five times	TA rinsing significantly reduced the number of samples covered by bacteria (2.8 ± 1). Salivary bacterial viability decreased to (47 ± 6) after 1 min of TA rinsing. Antimicrobial effect decreased after 30 min (64 ± 22) and returned to negative control values after 2 h (75 ± 13).
Schestakow et al. (2022) [60]	1% TA	In situ and ex vivo	Enamel from bovines worn by volunteers as maxillary splints	Rinsed with TA every 25 min	TA reduced calcium ion release in samples (1.09 ± 0.73) compared to the control (3.64 ± 2.45). TA-treated films had a higher electron density than the control, with a porous and sparse ultramicrostructure appearance.
Zhen et al. (2022) [61]	TS@NaF (10% TA)	In vitro and in vivo	Bovine enamel, rodent caries model	Demineralized enamel immersed in TS@NaF, TS@NaF hydrogels applied to rodent molars for caries prevention	Tensile bond strength of TS@NaF (67.18 ± 6.64 kPa) was significantly higher than that of other groups. Fluoride ion release quickly increased to 866.04 ± 105.66 µg/cm^2^ in the first 24 h, stabilizing at 1093.38 ± 95.95 µg/cm^2^ by day 7. No significant cytotoxicity was observed. Spherical CaF_2_ deposits formed a dense and consistent coating on the enamel. In vivo experiments showed that the total carious scores in the TS@NaF group (6.0% ± 3.0%) were significantly lower than those in the varnish group (10.0% ± 2.0%) and control group (23.0% ± 3.0%; *n* = 6; *p* < 0.01).
Kong et al. (2022) [62]	1.7% TA	In vitro	Human dentin	Demineralized dentin sections and collagen membranes immersed in TA for 2 h	Collagen film crosslinking degree after TA pretreatment was 41.28 ± 1.52. TA-crosslinked self-assembled collagen films exhibited a tighter arrangement with a dense multi-network. Interfacial energy of the mineralization system was reduced from 10.59 mJ/m^2^ to 10.59 mJ/m^2^ through crosslinking with TA. Measurements of TA-type dentin were 19.1 ± 1.12 GPa and 0.68 ± 0.06 GPa, close to healthy dentin measurements (21.7 ± 2.45 GPa and 0.9 ± 0.15 GPa).
Selvaraj et al. (2024) [63]	TA-AuNP (0.5% TA)	In vitro	*S. mutans*	TA-AuNP added to BHI agar plate of bacterial biofilms	Under streptomycin, TA, TA-AuNP, and cAuNP, the diameter of *S. mutans* was 21 ± 0.5 mm, 21 ± 1.0 mm, 25 ± 1.5 mm, and 33 ± 2 mm, respectively. Inhibition rates of TA-AuNPs in the biofilm were 1xMIC (4 μg/mL) 11%, 2xMIC (8 μg/mL) 57%, 3xMIC (12 μg/mL) 94%, and 4xMIC (16 μg/mL) 100%, respectively.

TA-F, tannic acid–fluoride mixture; SAP-TA, salivary-acquired pellicle bioinspired tannic acid; TS@NaF, tannic acid–silk fibroin–sodium fluoride; TA-AuNP, tannic-acid-mediated biosynthesized gold nanoparticles; CaF_2_, calcium fluoride; SDF, silver diamine fluoride, *S. mutans*, *Streptococcus mutans*.

**Table 4:** Summary of applications of TA and its derivatives in prosthesis and implant coating.

Studies	Treatment	Study Type	Subject	Assessment	Key Outcomes
Yang et al. (2017) [64]	SAP-TA	In vitro	HA, MG63, and *S. mutans*	Coated with SAP-TA for 24 h	Average roughness of SAP 3-TA-coated HA surface (88 ± 27 nm) was lower than that of bare HA (125.8 ± 25 nm). Saturation adsorption of 1.5 mg/50 mg HA powder with monolayer adsorption. Water contact angle of SAP 3-TA-coated HA (14.2°) was lower than that of bare TA (58°). OD 600 values for SAP 3-TA, SAP-coated HA, and bare HA were 0.336 ± 0.076, 0.65 ± 0.073, and 0.71 ± 0.08, respectively. Low cytotoxicity.
Weber et al. (2019) [65,66]	Siaq-TA (0.1% TA)	In vitro	SiO_2_, Au, HA, and Ti	Coated with Siaq-TA for 24 h	After 0.5 h, TA coating density was 2830 kg/m^3^, decreasing to 2550 kg/m^3^ after 1 h and 2060 kg/m^3^ after 2 h. Hydration of the TA layer was low in the first 30 min, increasing to 30% after 1 h. During oxidation at pH 6.8, more TA was available for coating deposition, achieving a uniform thickness of 266 ± 2 nm.
Steffi et al. (2019) [19]	0.2% TA	In vitro	Ti, RAW 264.7, and preosteoclast cells	Coated with TA overnight	TRAP activity decreased. ROS production reduced. Well biocompatibility.
Geissler et al. (2019) [28]	0.01% TA	In vitro	Ti, human osteoblasts, and *S. aureus*	Coated with TA for 2 or 24 h	mRNA levels of COL1A1, IL6, and ALP on TA-coated surfaces were reduced. Cytotoxicity increased compared to unmodified titanium control surfaces. All groups showed decreased OD compared to the control group, with the lowest CFU observed after 24 h of TA treatment.
Iqbal et al. (2020) [20]	TA/COL (0.01% TA)	In vitro	SiO_2_, hGFs, and *S. aureus*	Coated with TA/COL using the LBL technique (5 min deposition)	Release rate was highest in the first hour, stabilizing thereafter. After 72 h, TA release from the terminal citrate membrane was twice that of the acetate membrane (13.2 ± 1.3 μg/mL vs. 5.9 ± 1.2 μg/mL). Antimicrobial performance of TA/COL citrate membrane was unrelated to total TA release, which was far below the minimum inhibitory concentration. No cytotoxicity.
Li et al. (2020) [67]	TA-GO/Lys (0.1% TA)	In vitro	Glass slides, silicon wafers, and quartz plates, DPSCs, *E. coli*, and *S. aureus*	Coated with GO-Lys-TA using the LBL technique (10 min deposition)	The thickest coating obtained at pH 6.5 reached up to 210 nm, higher than others (<80 nm). Killing rate against *E. coli* was 90%, higher than that against *S. aureus*. No cytotoxicity observed. Increased expression of osteogenic genes (Runx2 and ALP).
Dong et al. (2021) [68]	pTA (2% TA)	In vitro	Ti, hGFs, l, *E. coli*, and *S. aureus*	Coated with the solution and irradiated by UV at 285 nm for 4 h	Hydrophilicity and stability increased. Nonspecific protein adsorption prevented. Bacterial activity inhibited. Macrophage polarization to M2 promoted, M1 inhibited. Well biocompatibility achieved.
Wang et al. (2021) [69]	TA@HA/Lys (0.5% TA)	In vitro and in vivo	Cover glass, silicon wafer, and titanium rod, DPSCs, MC3T3-E1, *E. coli*, *S. aureus*, and New Zealand rabbits	Coated with TA@HA/Lys using the LBL technique (10 min deposition), implant inserted into rabbit femoral condyle	Bacterial activity inhibited. H_2_O_2_ stimulation resisted. Rapid adhesion and long-term proliferation of MC3T3-E1 and DPSCs promoted. Bone formation enhanced in vivo. Well biocompatibility achieved.
Weber et al. (2022) [70]	0.1% TA	In vitro	Ti, hGFs	Coated with TA for 24 h	Complement and coagulation systems triggered. Pro-inflammatory cytokines expression reduced. Intracellular ROS in hGFs under oxidative stress reduced. No significant anti-inflammatory effect in hGFs stimulated by LPS and IL-1β Well biocompatibility.
Weber et al. (2022) [71]	0.1% TA	In vitro	Ti, *C. albicans*	Coated with TA for 24 h	Protein adsorption inhibited. No significant inhibition of *C. albicans*.
Kim et al. (2022) [72]	CHX-loaded TA-PEG (0.5% TA)	In vitro	PMMA, MC3T3-E1	Coated with CHX-loaded TA-PEG for 10, 20, 40, and 90 min	Average water contact angle (65.1°) was lower than that of untreated samples (75.2°). Coating thickness was uniform (average of 7.8 μm). Increasing PEG molecular weight with the same concentration of TA made the TA-PEG coating harder. Addition of 0.3 and 0.5 mg/mL CHX resulted in coatings with CHX loads of 108.2 μg and 183.2 μg, respectively, releasing 1.69 μg/mL and 2.89 μg/mL of drug. Well biocompatibility.
Liu et al. (2022) [73]	TA-nHA-PEG (0.2% TA)	In vitro and in vivo	Ti, BMSCs, *S. aureus*, *E. coli*, and rat model	Coated with TA-nHA-PEG using the LBL technique (4 h deposition), rod implanted in rat femur	Ti-HP showed lower *E. coli* attachment (4.89 ± 0.63 log) than Ti (33.62 ± 2.14 log). Lower *S. mutans* attachment (8.50 ± 2.54 log). ALP expression was doubled compared to Ti. Significant upregulation of osteogenic genes Alp, Ocn, and Runx2. Calcified nodules (0.96 ± 0.04) were significantly higher than Ti (0.09 ± 0.01). In vivo experiments showed BV/TV (Ti: 11.00 ± 7.02%, Ti-HP: 42.75 ± 6.24%), Tb.Th (Ti: 106.40 ± 22.61 μm, Ti-HP: 156.70 ± 27.20 μm), and Tb.Sp (Ti: 359.10 ± 30.51 μm, Ti-HP: 202.00 ± 35.61 μm). Low cytotoxicity.
Ren et al. (2022) [74]	TA/Sr^2+^ (1:1)	In vitro and in vivo	BMSCs, RAW264.7, and rat model	Cells cultured and treated with TA/Sr^2+^ (1 h), membrane inserted into rat alveolar bone	Differentiation of BMSCs into osteoblasts promoted. ROS release inhibited. Macrophage M1 polarization inhibited, M2 promoted. Bone regeneration enhanced.
Li et al. (2023) [75]	TA-Ce-Mino (0.4% TA)	In vitro and in vivo	Ti, PEEK, hGFs, and rat model	Immersed in TA-Ce-Mino and vortexed for 30 s, Ti disk inserted into rat model	Differentiation of BMSCs into osteoblasts promoted. ROS release inhibited. Macrophage M1 polarization inhibited, M2 promoted. VEGF-mediated angiogenesis and tissue regeneration enhanced. Well biocompatibility.
Shen et al. (2023) [76]	CS-GA/TAMP (40% TA)	In vitro and in vivo	Ti, hGFs, *P. gingivalis*, *F. nucleatum*, and pig model	Hydrogel samples soaked in lysozyme solution for 21 days, hydrogels injected in pig mandible around implant and exposed to bacterial suspensions	CS-GA hydrogels exhibited high lap shear strength (82.39 ± 11.58 KPa). CS-GA/TAMP 2 showed 68.61% and 83.11% antibacterial rates against *P. gingivalis* and 66.60 ± 7.51% and 83.48 ± 6.85% against *F. nucleatum* at different powers. Low cytotoxicity.
Zhao et al. (2023) [77]	TA-OGP@(RGD)*n* (0.1% TA)	In vitro and in vivo	Ti, MC3T3-E1, and rat model	Coated with TA-OGP@(RGD)n using the LBL technique (20 min deposition), Ti implants inserted into rat model	ROS balance regulated. Osteogenic differentiation and extracellular matrix mineralization promoted. Osteogenesis and bone integration enhanced. Well biocompatibility.

Siaq-TA, silicic acid–tannic acid; TA/COL, tannic acid–collagen; TA-GO/Lys, tannic acid–graphene oxide–lysozyme; pTA, poly tannic acid; TA@HA/Lys, tannic acid–hydroxyapatite–lysozyme; CHX-loaded TA-PEG, chlorhexidine acetate–tannic acid–polyethylene glycol; TA-nHA-PEG, tannic acid–inorganic hydroxyapatite nanoparticles–organic polyethylene glycol; TA/Sr^2+^, tannic acid–strontium ion; TA-Ce-Mino, tannic acid–cerium–minocycline; CS-GA/TAMP, gallic acid–grafted chitosan–tannic acid miniaturized particles; TA-OGP@(RGD)n, tannic acid–osteogenic growth peptide–arginine–glycine–aspartic acid; MG63, human osteoblast-like cell line; HA, hydroxyapatite; SiO, silicon dioxide; Au, aurum; Ti, titanium; RAW264.7, mouse macrophage cell line; TRAP, tartaric acid–resistant acid phosphatase; ROS, reactive oxygen species; COL1A1, collagen type I alpha 1; ALP, alkaline phosphatase; OC, osteocalcin; IL-6, interleukin-6; LBL, layer-by-layer; *E. coli*, *Escherichia coli*; *S. aureus*, *Staphylococcus aureus*; UV, ultraviolet; BSA, bovine serum albumin; H_2_O_2_, hydrogen peroxide; MC3T3-E1, mouse osteoblast cell line; LPS, lipopolysaccharide; IL-1β, interleukin-1 beta; *C. albicans*, *Candida albicans*; PMMA, polymethyl methacrylate; CHX, chlorhexidine acetate; BMSCs, bone marrow stem cells; PEEK, polyetheretherketone; *P. gingivalis*, *Porphyromonas gingivalis*; *F. nucleatum*, *Fusobacterium nucleatum*; VEGF, vascular endothelial growth factor.

**Table 5:** Summary of applications of TA and its derivative in periodontal and mucosal disease treatment.

Studies	Treatment	Study Type	Subject	Assessment	Key Outcomes
Homer et al. (1990) [78]	0.000001%, 0.00001%, 0.0001%, 0.001%, and 0.01% TA	In vitro	*B. gingivalis*, *B. intermedius*, and *T. denticola*	Bacteria strains cultivated with TA	0.0001–0.001% TA inhibited enzyme activities of *B. gingivalis* and *T. denticola* by 50%. 0.001–0.01% TA inhibited enzyme activities by 90%. 0.00001–0.0001% TA inhibited enzyme activities of *B. intermedius* by 50%.
Darvin et al. (2015) [79]	3.4–17% TA	In vitro	GSCC	TA applied to cells for 24–48 h, cell cycle and pathways examined	Cell cycle arrest and apoptosis induced in GSCC. Higher concentration led to stronger inhibition. Primarily through Jak2/STAT3 signaling pathway regulation.
Sheng et al. (2018) [80]	0.002% and 0.008% TA	In vitro	Human normal OLK and oral cancer cells	Cells treated with TA for 48 h after doxorubicin induction.	Doxorubicin-induced cytotoxicity reduced in keratinocytes. No reduction in anticancer efficacy of doxorubicin.
Shahbazi et al. (2020) [81]	PVA-TA, PATA (50% TA)	In vitro	Customized wearable holder, *S. aureus*	Hydrogel performance tested in drug delivery device	Water content of PATA remained stable (55–70%). Good mechanical recovery after 240 s. Fracture gaps filled after 20 s. PATA can stretch up to 30,000% of its original length. Exhibited ultimate stress (35–55 kPa). Reduced ROS and TNF-α, showing low cytotoxicity.
Lengert et al. (2021) [82]	AgNPs-TA (0.1% TA)	In vivo	Rat lower jaw anterior incisors region	Hydrogel capsule suspension applied to the rat model	Gingival perfusion correction efficiency improved. Neurogenic and myogenic tone of small arteries and precapillary sphincters improved. Blood outflow parameters improved.
Zhu et al. (2022) [23]	TA-SF-DP, TSD (10% TA)	In vitro and in vivo	Porcine buccal mucosa, rat tail truncation model, and coronal plane through extraction socket	Hydrogel tested in vitro and applied to animal models	Wet adhesion properties achieved. Drug release controlled. Strong hemostatic effect. Inflammatory gene expression (COX-2, IL-1β, TNF-α) downregulated. Inflammatory infiltration reduced.
Liu et al. (2022) [24]	CS-GOx-TA (0.2%, 0.4%, 0.6%, 0.8%, and 1% TA)	In vitro	MC3T3-E1, RAW264.7, and *P. gingivalis*	Hydrogel tested in vitro	Mechanical properties improved with higher concentrations. NO, IL-6, and TNF-α production inhibited. Bacterial activity inhibited. Glucose sensitivity achieved. Well biocompatibility.
Ding et al. (2022) [83]	isoG-TA (TA: 1/10 of isoG molar)	In vitro and in vivo	Mice anti-OLK model	Hydrogel tested in vitro and applied to the animal model	Strong wet adhesion properties. OLK proliferation inhibited. Dysplastic oral keratinocytes proliferation inhibited.
Zhao et al. (2022) [84]	spheroid@[Fe^3+^-TA] (4% TA)	In vitro	PDLSCs, *P. gingivalis*	PDLSCs spheroid coated with Fe^3+^-TA coordination network	Bacterial activity inhibited. Inflammatory factor release (IL-6, IL-10) suppressed. ROS regulated. Well biocompatibility.
Zhu et al. (2022) [85]	GNT (7.5%, 15% and 30% TA)	In vitro and in vivo	*S. aureus*, *E. coli*, rat and rabbit full-thickness oral mucosa model	Hydrogel tested in vitro and applied to the animal model	Strong wet adhesion properties. Excellent tensile properties. Hemostatic effect achieved. Bacterial activity inhibited. Inflammatory factors (TNF, IL-17) suppressed. Tissue-repair capability achieved.
Shi et al. (2022) [86]	G-TA@Mn^2+^	In vitro and in vivo	DCs/macrophages, mice anti-OLK model	Hydrogel tested in vitro and applied to the animal model	Gel group had a lower OSCC incidence (8%) compared to NC group (24%). No cases of severe dysplasia in the gel group (0%) vs. 29% in the NC group. Lower percentage of high-grade dysplasia in the gel group. Well biocompatibility.
Cheng et al. (2023) [87]	SF/TA (10% TA)	Ex vivo	NIH3T3, *S. aureus*, *E. coli*, and rat oral mucosa model	Patch applied to rat oral mucosa for 5 min	Cumulative drug release reached 88.37% within 7 days. Strong adhesion (37.74 kPa) about seven times that of commercial oral patches (<5 kPa). Well biocompatibility.
He et al. (2023) [88]	PGA/TA-NPs (0.3% TA)	In vitro and in vivo	RAW264.7, L929, *S. aureus*, *E. coli*, and rat oral ulcer model	Drug delivery system tested in vitro and applied to the rat model	ROS scavenged efficiently. Bacterial activity inhibited. High and stable drug release rate achieved. Strong tissue-healing effect. Well biocompatibility.
Liu et al. (2023) [89]	ZPTA-G/HMA (0.02% TA)	In vitro and in vivo	RAW264.7, *S. aureus*, *E. coli*, *C. albicans*, and rat oral ulcer model	Hydrogel tested in vitro and applied to the animal model	Bond strength increased from 8.45 ± 1.473 kPa to 46.25 ± 3.425 kPa. Compressive strength: 72.53 ± 7.99 kPa. Rat tail experiment showed enhanced BSA adsorption. Adjustable gelation time increased from 120 s to 240 s. Antibacterial performance: *E. coli* 92.27%, *S. aureus* 93.27%, and *C. albicans* 82.49%. Anti-inflammatory effects: TNF-α, IL-6, and IL-1β levels reduced by approximately 38.47%, 41.62%, and 43.70%. Well biocompatibility.

PATA, polyvinyl alcohol–tannic acid; AgNPs-TA, silver nanoparticles–tannic acid; TA-SF-DP, TSD, tannic acid–silk fibroin–diclofenac potassium; CS-GOx-TA, chitosan–glucose oxidase–tannic acid; isoG-TA, isoguanosine–tannic acid; spheroid@[Fe^3+^-TA], periodontal ligament stem cells spheroid–iron(III) ion–tannic acid; GNT, gelatin methacrylate–nanoclay–tannic acid; G-TA@Mn^2+^, guanosine–tannic acid–manganese ion; SF/TA, silk fibroin/tannic acid; PGA/TA-NPs, polyglutamic acid–tannic acid–nanoparticles; ZPTA-G/HMA, zinc oxide–tannic acid–methacrylate gelatin–methacrylate hyaluronic acid; *B. gingivalis*, *Bacteroides gingivalis*; *B. intermedius*, *Bacteroides intermedia*; *T. denticola*, *Treponema denticola*; GSCC, gingival squamous cell carcinoma; Jak2/STAT3, Janus kinase 2/signal transducer and activator of transcription 3; OLK, oral keratinocytes; COX-2, cyclooxygenase-2; TNF-α, tumor necrosis factor-alpha; NO, nitric oxide; PDLSCs, periodontal ligament stem cells; IL-10, interleukin-10; IL-17, interleukin-17; DCs, dendritic cells; NIH3T3, mouse embryonic fibroblastic cell line; L929, murine fibroblast cell line.

**Table 6:** Summary of applications of TA and its derivatives in endodontic treatment optimization.

Studies	Treatment	Study Type	Subject	Assessment	Key Outcomes
Bitter (1989) [25]	25% TA	In vitro	Human teeth	Root canal preparation and irrigation with H_2_O_2_ and NaClO, followed by TA irrigation for 10–90 s	TA rinsing for 10 s, 15 s, 20 s, and 90 s showed improved cleaning with longer times.
Raiden et al. (1997) [90]	25% TA	In vitro	Human teeth	Root canal cleaning with TA for 20 s, followed by dental cement filling and immersion in Indian ink	Microleakage before root canal filling was lower in the TA group (0.86 ± 1.32) compared to the control group (2.23 ± 1.39).
Raiden et al. (1998) [91]	25% TA	In vitro	Human teeth	Root canal cleaning with TA for 20 s, followed by dental cement filling and dowel space preparation	Removal time for root canal materials after 20 s of TA rinsing (54 ± 20 s) was longer than without TA (48 ± 21 s) or with chloroform first (31 ± 14 s). Cold lateral condensation required the shortest time (20 ± 13 s).
Yoshikawa et al. (1998) [92]	TA-α-TCP (5% TA)	In vitro	Human teeth	Root canal filled with TA-α-TCP	Solidification time: 8.5–9.0 min in the treatment group (average 8.8 ± 0.3 min) vs. 45–65 min in the control group (average 55.8 ± 9.8 min). Dye penetration was 1.2 ± 1.1 mm in the treatment group vs. 4.1 ± 2 mm in the control.
Yoshikawa et al. (2000, 2003) [93,94]	TA-α-TCP (5% TA)	In vitro and in vivo	Rat teeth	TA-α-TCP placed in periapical area of mechanical injury	The treatment group showed many foreign body giant cells and macrophages; the control group had severe inflammation with neutrophil aggregation (80%). After 3 weeks, the treatment group showed 80% alveolar bone reconstruction and reduced acute inflammation.
Yoshikawa et al. (2001) [95]	TA-α-TCP (5% TA)	In vitro	Rat teeth	TA-α-TCP positioned over residual pulp after molar pulp cutting	Material hardening facilitated. Phosphorus ion release promoted. Intrapulpal hard-tissue formation induced.
Nakamura et al. (2011) [96]	HY (20% TA)	In vitro	Rat clonal dental pulp cell line RPC-C2A	Cells cultured with cement extract for 24 h	After 3 h, Cox-2 mRNA expression significantly increased, then gradually decreased. PGE2 levels suppressed after 12–24 h. ATP content significantly increased after 24 h.
Christopher et al. (2016) [97]	10% TA	In vitro	Human teeth	Root canal preparation followed by TA irrigation for 10 min and obturation	Maximum canal wall penetration in the TA group (617.67 ± 1.55 mm) was greater than that in the non-TA group (466.66 ± 9.18 mm).
Kharouf et al. (2021) [98]	MTA-TA (6%, 12.5%, 18.75%, and 25% TA)	In vitro	MTA	MTA-TA preparation and performance examined	Time to completely mix 5 mL drops: MTA 100%: 45 ± 5 s; MTA 94%: 30 ± 3 s; MTA 87.5%: 13 ± 2 s; MTA 81.25%: 9 ± 1 s; MTA 75%: 0.5 ± 0.2 s.
Wu et al. (2022) [99]	CSC-TA (1%, 5%, and 10% TA)	In vitro	MG63, *E. coli*, and *S. aureus*	TA mixed with bioactive calcium silicate as liquid phase	TA reduced radial tensile strength (0.9 MPa) compared to the control (2.5 MPa). Antioxidant activity ranged from 84 to 88%, with no statistical difference from control. After 12 h, 1%, 5%, and 10% TA cement showed *E. coli* inhibition rates of 64%, 69%, and 72%, respectively, and similar trends were observed for *S. aureus*. Well biocompatibility.
Louvrier et al. (2022) [100]	PLA/PCL-TA (particles)	In vitro	DPSCs	PLA/PCL-TA inserted into biomimetic bioreactor mimicking human tooth root canal	After 78 h, the average cell count outside collagen gel was 844 ± 94 μm, with a migration distance of 502.7 ± 19.6 μm. After 1 week, the migration distance was 1000 μm, and after 3 weeks, it was 2400 μm. Well biocompatibility.
Zhou et al. (2024) [101]	HPCH/TA (7.5 mg/mL)	In vitro and in vivo	hDPCs, *S. mutans*, *E. faecalis*, and rat model	HPCH/TA hydrogel tested in vitro and evaluated as pulp capping material in vivo	HPCH hydrogel compression modulus: 1.5 ± 0.15 kPa; HPCH/TA hydrogel: 1.6 ± 0.17 kPa. Treated cells showed significantly reduced p65 nuclear translocation. Anti-inflammatory activity may occur via NF-κB pathway inhibition. Complete reparative dentin formation was observed under pulp capping material. IL-1β, IL-6, and TNF-α mRNA and protein expression decreased. Well biocompatibility.

TA-α-TCP, tannic acid–alpha–tricalcium phosphate; MTA-TA, mineral trioxide aggregated–tannic acid; CSC-TA, calcium silicate-based cement–tannic acid; PLA/PCL-TA, polylactic acid/polycaprolactone–tannic acid; HPCH/TA, hydroxypropyl chitin/tannic acid, RPC-C2A, rat clonal dental pulp cell line; PGE2, prostaglandin E2; MTA, mineral trioxide aggregate; CSCs, calcium silicate-based cement system; *E. faecalis*, *Enterococcus faecalis*.

**Table 7:** Summary of applications of TA and its derivatives in materials for public oral health.

Studies	Treatment	Study Type	Application Type	Subject	Assessment	Key Outcomes
Nordbö et al. (1983) [26]	0.006%, 0.05%, 0.1%, and 0.2% TA	In vitro and in vivo	Dental staining	Acrylic resin surfaces	Plaque formation on acrylic resin, soaked in TA	TA alone did not cause discoloration. 0.05% CH and 0.05% TA turned brown. 0.2% CH and 0.2% TA turned yellowish brown. 0.2% TA and 20 mM FeCl_3_ turned bluish black. 0.1% TA and 10 mM Fe^3+^ turned blue. 0.006% TA and 10 mM Fe^3+^ turned brown.
Kageyama et al. (1985) [102]	TA-UA (5% TA)	In vitro	Observing ultrastructural carbohydrates	Odontoblast, predentin, and dentin matrix of rat	Observing ultrastructural carbohydrates within cells and microscopic structures	Collagen fibers in anterior dentin were finer (about 50 nm) than those in calcified dentin (about 100 nm). Diameter increased as anterior dentin transformed into calcified dentin.
Takagi et al. (1989) [103]	TA-UA (5% TA)	In vitro	Observing ultrastructural cytochemical properties	Periodontal ligament of Alligator mississippiensis	Observing ultrastructural cytochemical properties of elastic elements in the periodontal ligament	Diameter of large and small bundles of periodontal ligament microfibrils: 12.0 ± 2.0 nm and 12.0 ± 2.2 nm, respectively. Larger dye deposits (6–22 nm) were localized in microfibrils, while smaller reactive deposits (4–7 nm) persisted in collagen-associated matrix materials.
Ishizeki et al. (1990) [104]	2% TA	In vitro	Fixing	AMF of mouse tooth germs	Microstructures examined by fixation with a substance containing TA	Electron-dense particles deposited on both surfaces of the basement membrane and AMF. AMF closely related to TA-positive particles in the transparent layer. Microfilament lengths were approximately 0–5 μm, occasionally reaching 1–3 μm.
Kim et al. (1994) [105]	Acetone-TA (1% TA)	In vitro	Fixing	Ameloblastsin molar tooth germs of neonatal rats	Microstructures examined by fixation and freeze-substituted with a substance containing TA	After rapid freezing, newly formed enamel matrix formed a three-dimensional interconnected chain, with a diameter of about 10 nm. Initially, the secreted particles were larger, gradually increasing to electron-dense particles of 70–150 nm in diameter.
Joiner et al. (2004) [106]	1% TA	In vitro and in situ	Dental staining	HA surface	Ellipsometry used to measure the coloring effect of TA on dental acquired film	High adsorption of TA. Buffer and SDS washing solutions could not remove the salivary layer.
Haruyama et al. (2018, 2022) [107,108]	0.1% TA	In vitro	Dental staining	Bovine teeth	Enamel polished, leveled, and stained with FeCl_3_ and TA	Stain removal scores for electric toothbrush + whitening agent and electric toothbrush + fluoride agent were higher than those for electric toothbrush alone. No significant difference between the two whitening groups (*p* > 0.05). No significant differences at 5, 10, and 20 min (*p* > 0.05).
Marquillas et al. (2020) [109]	8% TA	In vitro	Dental staining	Bovine teeth	TA solution used as dyeing model	Doubling TA increased the whitening effect by approximately 10% after 20 min.
Asghar et al. (2022) [110]	5%, 10%, and 15% TA	In vitro	Cleaning agent	Bovine teeth	TA used to modify SDF	Compared to 30% SDF, TA15 (39.91%) and TA10 (31.13%) had the highest staining rates. At 5%, TA-modified SDF reduced staining potential by 23% and 20.5% compared to calbiochem-modified SDF.
Cen et al. (2023) [111]	TA-CPC (0.5% TA)	In vitro	Cleaning agent	Orthodontic aligners	Aligners stained by coffee and immersed in the complex	Sample tensile breaking stress was 33.3 ± 1.63 MPa, not significantly different from samples immersed in water (33.1 ± 2.35 MPa). *S. aureus* and *E. coli* colonies shrank, with biofilm coverage reduced to 2.26% ± 0.21%. TA-CPC immersion group showed a color change value of 2.846 ± 0.55, significantly lower than that of deionized dH_2_O (10.26 ± 0.04), indicating a good cleaning ability. Well biocompatibility.

TA-UA, tannic acid–uranyl acetate; Acetone-TA, acetone–tannic acid; TA-CPC, tannic acid–cetylpyridinium chloride; CH, chlorhexidine; FeCl_3_, iron(III) chloride; AMF, aperiodic microfibrils; SDS, sodium dodecyl sulphate.

## Data Availability

The original data presented in the study are openly available in the references cited.

## Supplementary Materials

The following supporting information can be downloaded at: https://www.mdpi.com/article/10.3390/bioengineering12050438/s1, Table S1: Derivative sequence of keywords and MeSH terms for search strategy; Table S2: Search strategy employed for each database; Table S3: Quality assessment in the analyses.

## Abbreviations

Abbreviation	Meaning
TA	Tannic acid
IL	Interleukin
TNF	Tumor necrosis factor
DH	Dentin hypersensitivity
HY	Tannin–fluoride
FTLA	Fluoride–tannic acid–lanthanum–apatite
SF	Silk fibroin
GIC	Glass ionomer cement
ALP	Alkaline phosphatase
PA	Phosphoric acid
SDF	Silver diamine fluoride
ROS	Reactive oxygen species
LPS	Lipopolysaccharide
GGs	Gallic acid-derived pyruvate glucosides
RCT	Randomized controlled trial
