# Supplementary material for: Unleashing the Potential of Tannic Acid in Dentistry: A Scoping Review of Applications
Source: Bioengineering (Basel). 2025 Apr 22;12(5):438. doi: 10.3390/bioengineering12050438 (PMC12109371; doi:10.3390/bioengineering12050438)
Supplement: Supplementary file 1 [file bioengineering-12-00438-s001.zip › Supplementary table.pdf]

Supplementary Table 1. Derivative sequence of keywords and MeSH terms for search strategy

**Tannic Acid**	**Dentistry**		
Gallotannin	Dental Instruments	Dental Care	Diagnosis, Oral
Gallotannic Acid	Economics, Dental	Dental Debonding	Electrogalvanism, Intraoral
(2S,3R,4S,5R,6R)-3,4,5-tris(3,4-dihydroxy-5-(3,4,5-trihydroxybenzoyloxy)benzoyloxy)-6-((3,4-dihydroxy-5-(3,4,5-trihydroxybenzoyloxy)benzoyloxy)methyl)oxan-2-yl 3,4-dihydroxy-5-(3,4,5-trihydroxybenzoyloxy)benzoate	Education, Dental	Dental Equipment	Endodontics
	History of Dentistry	Dental Health Surveys	Esthetics, Dental
	Legislation, Dental	Dental High-Speed Technique	Infection Control, Dental
	Air Abrasion, Dental	Dental Occlusion	Jaw Relation Record
	Anesthesia, Dental	Dental Pins	Surgery, Oral
	Dental Atraumatic Restorative Treatment	Dental Polishing	Technology, Dental
	Dental Bonding	Dental Stress Analysis	Tooth Preparation
	Models, Dental	Orthodontics	Tooth Remineralization
	Mouth Rehabilitation	Pathology, Oral	Oral Medicine
	Myofunctional Therapy	Periodontics	Oral Surgical Procedures
	Odontometry	Preventive Dentistry	Prosthodontics

Supplementary Table 2. Search strategy employed for each database

**Database**	**Search strategy**
PubMed<br>N = 309	#1 (((Tannic Acid[MeSH Terms]) OR (Gallotannin[Title/Abstract])) OR (Gallotannic Acid[Title/Abstract])) OR (Gallotannic Acid (2S,3R,4S,5R,6R)-3,4,5-tris(3,4-dihydroxy-5-(3,4,5-trihydroxybenzoyloxy)benzoyloxy)-6-((3,4-dihydroxy-5-(3,4,5-trihydroxybenzoyloxy)benzoyloxy)methyl)oxan-2-yl 3,4-dihydroxy-5-(3,4,5-trihydroxybenzoyloxy)benzoate[Title/Abstract])<br>#2 ((((((((((((((((((((((((((((((((((((((dentistry[MeSH Terms]) OR (Dental Instruments[Title/Abstract])) OR (Economics, Dental[Title/Abstract])) OR (Education, Dental[Title/Abstract])) OR (History of Dentistry[Title/Abstract])) OR (Legislation, Dental[Title/Abstract])) OR (Air Abrasion,

	Dental[Title/Abstract])) OR (Anesthesia, Dental[Title/Abstract])) OR (Dental Atraumatic Restorative Treatment[Title/Abstract])) OR (Dental Bonding[Title/Abstract])) OR (Dental Care[Title/Abstract])) OR (Dental Debonding[Title/Abstract])) OR (Dental Equipment[Title/Abstract])) OR (Dental Health Surveys[Title/Abstract])) OR (Dental High-Speed Technique[Title/Abstract])) OR (Dental Occlusion[Title/Abstract])) OR (Dental Pins[Title/Abstract])) OR (Dental Polishing[Title/Abstract])) OR (Dental Stress Analysis[Title/Abstract])) OR (Diagnosis, Oral[Title/Abstract])) OR (Electrogalvanism, Intraoral[Title/Abstract])) OR (Endodontics[Title/Abstract])) OR (Esthetics, Dental[Title/Abstract])) OR (Infection Control, Dental[Title/Abstract])) OR (Jaw Relation Record[Title/Abstract])) OR (Models, Dental[Title/Abstract])) OR (Mouth Rehabilitation[Title/Abstract])) OR (Myofunctional Therapy[Title/Abstract])) OR (Odontometry[Title/Abstract])) OR (Oral Medicine[Title/Abstract])) OR (Oral Surgical Procedures[Title/Abstract])) OR (Orthodontics[Title/Abstract])) OR (Pathology, Oral[Title/Abstract])) OR (Periodontics[Title/Abstract])) OR (Preventive Dentistry[Title/Abstract])) OR (Prosthodontics[Title/Abstract])) OR (Surgery, Oral[Title/Abstract])) OR (Technology, Dental[Title/Abstract])) OR (Tooth Preparation[Title/Abstract])) OR (Tooth Remineralization[Title/Abstract])<br>#3 #1 AND #2
Cochrane<br>N=7	#1 MeSH descriptor: [Dentistry] explode all trees<br>#2 Dental Instruments<br>#3 Economics, Dental<br>#4 Education, Dental<br>#5 History of Dentistry<br>#6 Legislation, Dental<br>#7 Air Abrasion, Dental<br>#8 Anesthesia, Dental<br>#9 Dental Atraumatic Restorative Treatment<br>#10 Dental Bonding<br>#11 Models, Dental<br>#12 Mouth Rehabilitation<br>#13 Myofunctional Therapy<br>#14 Odontometry

#15 Dental Care
#16 Dental Debonding
#17 Dental Equipment
#18 Dental Health Surveys
#19 Dental High-Speed Technique
#20 Dental Occlusion
#21 Dental Pins
#22 Dental Polishing
#23 Dental Stress Analysis
#24 Orthodontics
#25 Pathology, Oral
#26 Periodontics
#27 Preventive Dentistry
#28 Diagnosis, Oral
#29 Electrogalvanism, Intraoral
#30 Endodontics
#31 Esthetics, Dental
#32 Infection Control, Dental
#33 Jaw Relation Record
#34 Surgery, Oral
#35 Technology, Dental
#36 Tooth Preparation
#37 Tooth Remineralization
#38 Oral Medicine
#39 Oral Surgical Procedures
#40 Prosthodontics

	#41 #1 or #2 or #3 or #4 or #5 or #6 or #7 or #8 or #9 or #10 or #11 or #12 or #13 or #14 or #15 or #16 or #17 or #18 or #19 or #20 or #21 or #22 or #23 or #24 or #25 or #26 or #27 or #28 or #29 or #30 or #31 or #32 or #33 or #34 or #35 or #36 or #37 or #39 or #39 or #40<br>#42 Tannic acid<br>#43 Gallotannin<br>#44 Gallotannic Acid<br>#45 #42 OR #43 OR #44<br>#46 #41 AND #45
Embase<br>N=107	#1 'dentistry'/exp OR 'dentistry'<br>#2 'dental medicine':ab,ti OR 'dental specialties':ab,ti OR 'dental specialty':ab,ti OR 'dental system':ab,ti OR 'occupational dentistry':ab,ti OR 'pathology, oral':ab,ti OR 'specialties, dental':ab,ti OR 'state dentistry':ab,ti OR 'dentistry':ab,ti<br>#3 'Dentistry':ab,ti OR 'Dental Instruments':ab,ti OR 'Economics, Dental':ab,ti OR 'Education, Dental':ab,ti OR 'History of Dentistry':ab,ti OR 'Legislation, Dental':ab,ti OR 'Air Abrasion, Dental':ab,ti OR 'Anesthesia, Dental':ab,ti OR 'Dental Atraumatic Restorative Treatment':ab,ti OR 'Dental Bonding':ab,ti OR 'Dental Care':ab,ti OR 'Dental Debonding':ab,ti OR 'Dental Equipment':ab,ti OR 'Dental Health Surveys':ab,ti OR 'Dental High-Speed Technique':ab,ti OR 'Dental Occlusion':ab,ti OR 'Dental Pins':ab,ti OR 'Dental Polishing':ab,ti OR 'Dental Stress Analysis':ab,ti OR 'Diagnosis, Oral':ab,ti OR 'Electrogalvanism, Intraoral':ab,ti OR 'Endodontics':ab,ti OR 'Esthetics, Dental':ab,ti OR 'Infection Control, Dental':ab,ti OR ‘Jaw Relation Record':ab,ti OR 'Models, Dental':ab,ti OR 'Mouth Rehabilitation':ab,ti OR 'Myofunctional Therapy':ab,ti OR 'Odontometry':ab,ti OR 'Oral Medicine':ab,ti OR 'Oral Surgical Procedures':ab,ti OR 'Orthodontics':ab,ti OR 'Pathology, Oral':ab,ti OR 'Periodontics':ab,ti OR 'Preventive Dentistry':ab,ti OR Prosthodontics':ab,ti OR 'Surgery, Oral':ab,ti OR 'Technology, Dental':ab,ti OR 'Tooth Preparation':ab,ti OR 'Tooth Remineralization':ab,ti<br>#4 #1 OR #2 OR #3<br>#5 tannic acid'/exp OR 'tannic acid'<br>#6 'Tannic Acid':ab,ti OR 'Gallotannin':ab,ti OR 'Gallotannic Acid':ab,ti OR '(2S,3R,4S,5R,6R)-3,4,5-tris(3,4-dihydroxy-5-(3,4,5-trihydroxybenzoyloxy)benzoyloxy)-6-((3,4-dihydroxy-5-(3,4,5-trihydroxybenzoyloxy)benzoyloxy)methyl)oxan-2-yl 3,4-dihydroxy-5-(3,4,5-trihydroxybenzoyloxy)benzoate':ab,ti<br>#7 #5 OR #6<br>#8 #4 AND #7

Scopus<br>N=39	#1 (TITLE-ABS-KEY ("dentistry") OR TITLE-ABS-KEY ("dental instruments") OR TITLE-ABS-KEY ("economics, dental") OR TITLE-ABS-KEY ("education, dental") OR TITLE-ABS-KEY ("history of dentistry") OR TITLE-ABS-KEY ("legislation, dental") OR TITLE-ABS-KEY ("air abrasion, dental") OR TITLE-ABS-KEY ("anesthesia, dental") OR TITLE-ABS-KEY ("dental atraumatic restorative treatment") OR TITLE-ABS-KEY ("dental bonding") OR TITLE-ABS-KEY ("models, dental") OR TITLE-ABS-KEY ("mouth rehabilitation") OR TITLE-ABS-KEY ("myofunctional therapy") OR TITLE-ABS-KEY ("odontometry") OR TITLE-ABS-KEY ("dental care") OR TITLE-ABS-KEY ("dental debonding") OR TITLE-ABS-KEY ("dental equipment") OR TITLE-ABS-KEY ("dental health surveys") OR TITLE-ABS-KEY ("dental high-speed technique") OR TITLE-ABS-KEY ("dental occlusion") OR TITLE-ABS-KEY ("dental pins") OR TITLE-ABS-KEY ("dental polishing") OR TITLE-ABS-KEY ("dental stress analysis") OR TITLE-ABS-KEY ("orthodontics") OR TITLE-ABS-KEY ("pathology, oral") OR TITLE-ABS-KEY ("periodontics") OR TITLE-ABS-KEY ("preventive dentistry") OR TITLE-ABS-KEY ("diagnosis, oral") OR TITLE-ABS-KEY ("electrogalvanism, intraoral") OR TITLE-ABS-KEY ("endodontics") OR TITLE-ABS-KEY ("esthetics, dental") OR TITLE-ABS-KEY ("infection control, dental") OR TITLE-ABS-KEY ("jaw relation record") OR TITLE-ABS-KEY ("surgery, oral") OR TITLE-ABS-KEY ("technology, dental") OR TITLE-ABS-KEY ("tooth preparation") OR TITLE-ABS-KEY ("tooth remineralization") OR TITLE-ABS-KEY ("oral medicine") OR TITLE-ABS-KEY ("oral surgical procedures") OR TITLE-ABS-KEY ("prosthodontics"))<br>#2 (TITLE-ABS-KEY ("tannic acid") OR TITLE-ABS-KEY ("gallotannin") OR TITLE-ABS-KEY ("gallotannic acid") OR TITLE-ABS-KEY ("(2s,3r,4s,5r,6r)-3,4,5-tris(3,4-dihydroxy-5-(3,4,5-trihydroxybenzoyloxy)benzoyloxy)-6-((3,4-dihydroxy-5-(3,4,5-trihydroxybenzoyloxy)benzoyloxy)methyl)oxan-2-yl 3,4-dihydroxy-5-(3,4,5-trihydroxybenzoyloxy)benzoate"))<br>#3 #1 AND #2
Web of Science<br>N=50	#1 "(((TS=(tannic acid)) OR TS=(Gallotannin)) OR TS=(Gallotannic Acid)) OR TS=((2S,3R,4S,5R,6R)-3,4,5-tris(3,4-dihydroxy-5-(3,4,5-trihydroxybenzoyloxy)benzoyloxy)-6-((3,4-dihydroxy-5-(3,4,5-trihydroxybenzoyloxy)benzoyloxy)methyl)oxan-2-yl 3,4-dihydroxy-5-(3,4,5-trihydroxybenzoyloxy)benzoate)"<br>#2 "(((((((((((((((((((((((((((((((((((((((TS=(Dentistry)) OR TS=(Dental Instruments)) OR TS=(Economics, Dental)) OR TS=(Education, Dental)) OR TS=(History of Dentistry)) OR TS=(Legislation, Dental)) OR TS=(Air Abrasion, Dental)) OR TS=(Anesthesia, Dental)) OR TS=(Dental Atraumatic Restorative Treatment)) OR TS=(Dental Bonding)) OR TS=(Models, Dental)) OR TS=(Mouth Rehabilitation)) OR TS=(Myofunctional Therapy)) OR TS=(Odontometry)) OR TS=(Dental Care)) OR TS=(Dental Debonding)) OR TS=(Dental Equipment)) OR TS=(Dental Health Surveys)) OR TS=(Dental High-Speed Technique)) OR TS=(Dental Occlusion)) OR TS=(Dental Pins)) OR TS=(Dental Polishing)) OR TS=(Dental Stress Analysis)) OR TS=(Orthodontics)) OR TS=(Pathology, Oral)) OR TS=(Periodontics)) OR TS=(Preventive Dentistry)) OR TS=(Diagnosis, Oral)) OR

TS=(Electrogalvanism, Intraoral)) OR TS=(Endodontics)) OR TS=(Esthetics, Dental)) OR TS=(Infection Control, Dental)) OR TS=(Jaw Relation Record)) OR TS=(Surgery, Oral)) OR TS=(Technology, Dental)) OR TS=(Tooth Preparation)) OR TS=(Tooth Remineralization)) OR TS=(Oral Medicine)) OR TS=(Oral Surgical Procedures)) OR TS=(Prosthodontics)"
#3 "#1 AND #2"

Supplementary Table 3. Quality assessment in the analyses

	Studies	Objective	Study design	Sample size calculation	Sample dimension	Sample randomization	Intervention methods	TA concentration description	Statistical methods	Results	Risk of bias
Dentin hypersensitivity management	Addy et al. (1987) [30]	Y	N	N	Y	N	Y	N	Y	Y	Moderate
	Yamaga et al. (1993) [31]	Y	N	N	Y	N	Y	Y	N	Y	Moderate
	Sabbak et al. (1998) [15]	Y	N	N	N	N	Y	Y	N	Y	High
	Mukai et al. (1998) [32]	Y	N	N	Y	N	Y	Y	N	Y	Moderate
	Tomiyama et al. (2004) [33]	Y	N	N	Y	N	Y	Y	Y	Y	Moderate
	Oh et al. (2015) [34]	Y	N	N	Y	N	Y	Y	N	Y	Moderate
	Li et al. (2020) [16]	Y	N	N	Y	N	Y	N	Y	Y	Moderate
	Gao et al. (2024) [35]	Y	Y	N	Y	N	Y	Y	Y	Y	Low
	A few articles were produced by the same team of researchers working on the topic. ([32]&[33])										
Bond strength improvement	Powis et al. (1982) [36]	Y	N	N	Y	N	Y	Y	N	Y	Moderate
	Prati et al. (1989) [37]	Y	N	N	Y	N	Y	Y	Y	Y	Moderate
	Bitter (1989) [38]	Y	N	N	N	N	Y	Y	N	Y	High
	Bitter (1990) [39]	Y	N	N	N	N	Y	Y	N	Y	High

	Okamoto et al. (1990) [40]	Y	N	Y	N	N	Y	N	N	Y	High
	Okamoto et al. (1991) [41]	Y	N	N	Y	N	Y	Y	N	Y	Moderate
	Prati et al. (1992) [21]	Y	N	N	Y	N	Y	Y	Y	Y	Moderate
	Natsir et al. (1999) [42]	Y	N	N	Y	N	Y	Y	N	Y	Moderate
	Kapoor et al. (2002) [43]	Y	N	N	Y	N	Y	Y	N	Y	Moderate
	Buchalla et al. (2007) [44]	Y	N	N	Y	N	Y	Y	Y	Y	Moderate
	Bedran-Russo et al. (2009) [22]	Y	N	N	Y	N	Y	Y	Y	Y	Moderate
	Pavan et al. (2010) [45]	Y	N	N	Y	N	Y	Y	Y	Y	Moderate
	Anil et al. (2015) [27]	Y	N	N	N	N	Y	Y	Y	Y	Moderate
	Alireza et al. (2017) [46]	Y	N	N	Y	N	Y	Y	Y	Y	Moderate
	Abdollahi et al. (2017) [47]	Y	N	N	Y	N	Y	Y	Y	Y	Moderate
	Cecchin et al. (2018) [48]	Y	N	Y	Y	N	Y	Y	Y	Y	Low
	Shafeie et al. (2022) [49]	Y	N	N	Y	N	Y	Y	Y	Y	Moderate
	Zheng et al. (2023) [50]	Y	Y	N	Y	N	Y	Y	Y	Y	Low
	A few articles were produced by the same team of researchers working on the topic. ([32]&[33])										
	Yu et al. (1993) [51]	Y	N	N	Y	N	Y	Y	Y	Y	Moderate
	Yu et al. (1995) [52]	Y	N	N	Y	N	Y	Y	N	Y	Moderate
Caries arrest	Yamaga et al. (1997) [53]	Y	Y	N	N	N	Y	Y	N	Y	Moderate
	Koide et al. (1997) [54]	Y	N	N	Y	N	Y	Y	Y	Y	Moderate
	Yee et al. (2009) [55]	Y	Y	Y	Y	N	Y	N	Y	Y	Low
	Yang et al. (2017) [56]	Y	Y	N	Y	N	Y	Y	N	Y	Moderate

	Hertel et al. (2017) [17]	Y	Y	N	Y	N	Y	Y	Y	Y	Low
	Huang et al. (2017) [18]	Y	N	N	Y	N	Y	Y	Y	Y	Moderate
	Xi et al. (2020) [57]	Y	Y	N	Y	N	Y	Y	Y	Y	Low
	Schestakow et al. (2020) [58]	Y	Y	N	Y	N	Y	Y	Y	Y	Low
	Schestakow et al. (2021) [59]	Y	Y	N	Y	N	Y	Y	Y	Y	Low
	Schestakow et al. (2022) [60]	Y	Y	N	Y	N	Y	Y	Y	Y	Low
	Zhen et al. (2022) [61]	Y	Y	N	Y	N	Y	Y	Y	Y	Low
	Kong et al. (2022) [62]	Y	N	N	Y	N	Y	Y	N	Y	Moderate
	Selvaraj et al. (2024) [63]	Y	N	N	Y	N	Y	Y	N	Y	Moderate
	A few articles were produced by the same team of researchers working on the topic. ([51]&[52]; [58]&[59]&[60])										
Implant and prosthesis coating	Yang et al. (2017) [64]	Y	N	N	Y	N	Y	N	Y	Y	Moderate
	Weber et al. (2019) [65]	Y	N	N	Y	N	Y	Y	N	Y	Moderate
	Weber et al. (2019) [66]	Y	N	N	Y	N	Y	Y	N	Y	Moderate
	Steffi et al. (2019) [19]	Y	N	N	Y	N	Y	Y	Y	Y	Moderate
	Geissler et al. (2019) [28]	Y	N	N	Y	N	Y	Y	Y	Y	Moderate
	Iqbal et al. (2020) [20]	Y	N	N	Y	N	Y	Y	N	Y	Moderate
	Li et al. (2020) [67]	Y	N	N	Y	N	Y	Y	Y	Y	Moderate
	Dong et al. (2021) [68]	Y	N	N	Y	N	Y	Y	Y	Y	Moderate
	Wang et al. (2021) [69]	Y	Y	N	Y	N	Y	Y	Y	Y	Low
	Weber et al. (2022) [70]	Y	N	N	N	N	Y	Y	Y	Y	Moderate
	Weber et al. (2022) [71]	Y	N	N	N	N	Y	Y	Y	Y	Moderate
	Kim et al. (2022) [72]	Y	N	N	Y	N	Y	Y	Y	Y	Moderate

	Liu et al. (2022) [73]	Y	Y	N	Y	N	Y	Y	Y	Y	Low
	Ren et al. (2022) [74]	Y	Y	N	Y	N	Y	N	Y	Y	Moderate
	Li et al. (2023) [75]	Y	Y	N	Y	N	Y	Y	Y	Y	Low
	Shen et al. (2023) [76]	Y	Y	N	Y	N	Y	Y	Y	Y	Low
	Zhao et al. (2023) [77]	Y	Y	N	Y	N	Y	Y	Y	Y	Low
	A few articles were produced by the same team of researchers working on the topic. ([65]&[66]&[70]&[71])										
Periodontal and mucosal inflammation control	Homer et al. (1990) [78]	Y	N	N	Y	N	Y	Y	Y	Y	Moderate
	Darvin et al. (2015) [79]	Y	N	N	Y	N	Y	Y	Y	Y	Moderate
	Sheng et al. (2018) [80]	Y	N	N	Y	N	Y	Y	Y	Y	Moderate
	Shahbazi et al. (2020) [81]	Y	N	N	Y	N	Y	Y	Y	Y	Moderate
	Lengert et al. (2021) [82]	Y	N	N	Y	N	Y	Y	Y	Y	Moderate
	Zhu et al. (2022) [23]	Y	Y	N	Y	N	Y	Y	Y	Y	Low
	Liu et al. (2022) [24]	Y	N	N	Y	N	Y	Y	Y	Y	Moderate
	Ding et al. (2022) [83]	Y	Y	N	Y	N	Y	N	Y	Y	Moderate
	Zhao et al. (2022) [84]	Y	N	N	Y	N	Y	Y	Y	Y	Moderate
	Zhu et al. (2022) [85]	Y	Y	N	Y	N	Y	Y	Y	Y	Low
	Shi et al. (2022) [86]	Y	Y	N	Y	N	Y	N	Y	Y	Moderate
	Cheng et al. (2023) [87]	Y	Y	N	Y	N	Y	Y	Y	Y	Low
	He et al. (2023) [88]	Y	Y	N	Y	N	Y	Y	Y	Y	Low
	Liu et al. (2023) [89]	Y	Y	N	Y	N	Y	Y	Y	Y	Low
	A few articles were produced by the same team of researchers working on the topic. ([23]&[85])										
Endodontic treatment optimization	Bitter (1989) [25]	Y	N	N	N	N	Y	Y	N	Y	High
	Raiden et al. (1997) [90]	Y	N	N	Y	N	Y	Y	Y	Y	Moderate
	Raiden et al. (1998) [91]	Y	N	N	Y	N	Y	Y	Y	Y	Moderate

	Yoshikawa et al. (1998) [92]	Y	N	N	Y	N	Y	Y	Y	Y	Moderate
	Yoshikawa et al. (2000) [93]	Y	Y	N	Y	N	Y	Y	Y	Y	Low
	Yoshikawa et al. (2001) [94]	Y	Y	N	Y	N	Y	Y	Y	Y	Low
	Yoshikawa et al. (2003) [95]	Y	N	N	Y	N	Y	Y	Y	Y	Moderate
	Nakamura et al. (2011) [96]	Y	N	N	Y	N	Y	Y	Y	Y	Moderate
	Christopher et al. (2016) [97]	Y	N	N	Y	N	Y	Y	Y	Y	Moderate
	Kharouf et al. (2021) [98]	Y	N	N	Y	N	Y	Y	Y	Y	Moderate
	Wu et al. (2022) [99]	Y	N	N	Y	N	Y	Y	Y	Y	Moderate
	Louvrier et al. (2022) [100]	Y	N	N	Y	N	Y	Y	Y	Y	Moderate
	Zhou et al. (2024) [101]	Y	Y	N	Y	N	Y	Y	Y	Y	Low
	A few articles were produced by the same team of researchers working on the topic. ([90]&[91], [92]&[93]&[94]&[95])										
	Nordbö ea al. (1983) [26]	Y	Y	N	Y	N	Y	Y	N	Y	Moderate
	Kageyama et al. (1985) [102]	Y	N	N	N	N	Y	Y	N	Y	High
Public oral health	Takagi et al. (1989) [103]	Y	N	N	Y	N	Y	Y	N	Y	Moderate
	Ishizeki et al. (1990) [104]	Y	N	N	N	N	Y	Y	N	Y	High
	Kim et al. (1994) [105]	Y	N	N	Y	N	Y	Y	N	Y	Moderate
	Joiner et al. (2004) [106]	Y	Y	N	Y	N	Y	Y	N	Y	Moderate

	Haruyama et al. (2018) [107]	Y	N	N	Y	N	Y	Y	Y	Y	Moderate
	Haruyama et al. (2022) [108]	Y	N	N	Y	N	Y	Y	Y	Y	Moderate
	Marquillas et al. (2020) [109]	Y	N	N	Y	N	Y	Y	Y	Y	Moderate
	Asghar et al. (2022) [110]	Y	N	N	Y	N	Y	Y	Y	Y	Moderate
	Cen et al. (2023) [111]	Y	N	N	Y	N	Y	Y	Y	Y	Moderate
	A few articles were produced by the same team of researchers working on the topic. ([107]&[108])										
